# Haplotype-resolved genome assembly and allele-specific gene expression in cultivated ginger

**DOI:** 10.1038/s41438-021-00599-8

**Published:** 2021-08-05

**Authors:** Shi-Ping Cheng, Kai-Hua Jia, Hui Liu, Ren-Gang Zhang, Zhi-Chao Li, Shan-Shan Zhou, Tian-Le Shi, Ai-Chu Ma, Cong-Wen Yu, Chan Gao, Guang-Lei Cao, Wei Zhao, Shuai Nie, Jing-Fang Guo, Si-Qian Jiao, Xue-Chan Tian, Xue-Mei Yan, Yu-Tao Bao, Quan-Zheng Yun, Xin-Zhu Wang, Ilga Porth, Yousry A. El-Kassaby, Xiao-Ru Wang, Zhen Li, Yves Van de Peer, Jian-Feng Mao

**Affiliations:** 1 Pingdingshan University, Henan Province Key Laboratory of Germplasm Innovation and Utilization of Eco-economic Woody Plant, Pingdingshan, 467000 Henan China; 2 Beijing Advanced Innovation Center for Tree Breeding by Molecular Design, National Engineering Laboratory for Tree Breeding, Key Laboratory of Genetics and Breeding in Forest Trees and Ornamental Plants, Ministry of Education, College of Biological Sciences and Technology, Beijing Forestry University, Beijing, 100083 China; 3 Ori (Shandong) Gene Science and Technology Co., Ltd, Weifang, 261000 Shandong China; 4 Pingdingshan Academy of Agricultural Sciences, Pingdingshan, 467000 Henan China; 5 Department of Ecology and Environmental Science, UPSC, Umeå University, Umeå, Sweden; 6 Département des Sciences du Bois et de la Forêt, Faculté de Foresterie, de Géographie et Géomatique, Université Laval Québec, Québec, QC G1V 0A6 Canada; 7 Department of Forest and Conservation Sciences, Faculty of Forestry, University of British Columbia, Vancouver, BC V6T 1Z4 Canada; 8 Department of Plant Biotechnology and Bioinformatics, Ghent University, Ghent, Belgium; 9 VIB Center for Plant Systems Biology, 9052 Ghent, Belgium; 10 Centre for Microbial Ecology and Genomics, Department of Biochemistry, Genetics and Microbiology Genetics, University of Pretoria, Private Bag X20, Pretoria, 0028 South Africa; 11 College of Horticulture, Academy for Advanced Interdisciplinary Studies, Nanjing Agricultural University, Nanjing, 210095 China

**Keywords:** Plant evolution, Gene expression, Gene regulation

## Abstract

Ginger (*Zingiber officinale*) is one of the most valued spice plants worldwide; it is prized for its culinary and folk medicinal applications and is therefore of high economic and cultural importance. Here, we present a haplotype-resolved, chromosome-scale assembly for diploid ginger anchored to 11 pseudochromosome pairs with a total length of 3.1 Gb. Remarkable structural variation was identified between haplotypes, and two inversions larger than 15 Mb on chromosome 4 may be associated with ginger infertility. We performed a comprehensive, spatiotemporal, genome-wide analysis of allelic expression patterns, revealing that most alleles are coordinately expressed. The alleles that exhibited the largest differences in expression showed closer proximity to transposable elements, greater coding sequence divergence, more relaxed selection pressure, and more transcription factor binding site differences. We also predicted the transcription factors potentially regulating 6-gingerol biosynthesis. Our allele-aware assembly provides a powerful platform for future functional genomics, molecular breeding, and genome editing in ginger.

## Introduction

At the dawn of civilization, spices were sought after as eagerly as gold and precious gems. Ginger (*Zingiber officinale*) was initially cultivated and utilized by Austronesian people more than 3000 years ago and was subsequently introduced to South India following Austronesian expansion^1^. The Latin name of the genus, *Zingiber*, is derived from the Greek word *zingiberis*, whose etymology can ultimately be traced to the Sanskrit word *srngaverram*, from *srngam* for “horn” and *vera* for “body”, describing the shape of the ginger rhizome^1^. Due to its unique flavor and popular appeal, ginger was finally brought to the Middle East and the Mediterranean by traders^1^. The spicy flavor of ginger rhizomes is conferred by a number of pungent compounds^2^, among which gingerols are the chemicals stimulating a spicy sensation on the tongue. Gingerols have different carbon chain lengths ranging from six to ten, among which 6-gingerol is the most abundant compound in the ginger rhizome^3^. The phenylpropanoid 6-gingerol has been reported to possess anticancer, antifungal, antiinflammation, antioxidant, and antiplatelet aggregation activities, among other biological properties^4–6^. Currently, ginger is an economically important and widely used spice and folk medicine worldwide. According to the Food and Agriculture Organization of the United Nations, the production of ginger had reached 2.78 million tons from a harvested area of 373,120 ha as of 2018, with 81.7% of the global production taking place in Asia (http://www.fao.org/faostat/en/#data/QC/). Despite the worldwide use of ginger, the genetic research and development efforts associated with it have not been commensurate with its importance.

As a true cultigen that no longer exists in the wild, ginger, unlike other species with a sexual reproductive mode, is sterile^7^. It is speculated that hybridization lies at the origin of contemporary cultivated ginger^1,8^. As a common approach during domestication, hybridization is carried out to transfer characteristics from two parents into their descendants, which will be selected when they show increased production or increased environmental tolerance. Such improved traits of descendants are usually due to increased genetic variation, brought about by mechanisms such as the differential expression of alleles at specific loci^9^. Indeed, the timing and duration of the expression of different alleles, as well as the quantities of their gene products, may differ substantially^10^, resulting in various phenotypic consequences^11^, providing important source material for artificial selection. High-quality, haplotype-resolved genome assembly and allele-specific gene expression data may provide further insights into the origin and evolution of traits specific to cultivated ginger.

Here, we report a chromosome-scale haplotype-resolved genome assembly of “Zhangliang” ginger, a landrace passed down from the Chinese Han dynasty for more than 2000 years, through asexual reproduction. This variety is now endemic to Lushan County, Pingdingshan, Henan Province, China, and is well known for its strong pungent flavor and richness in gingerol, ginger oil, and amino acids. Through a combination of Oxford Nanopore Technologies (ONT) sequencing and Hi-C (in vitro fixation of chromosomes) mapping, we generated a chromosome-scale assembly with a total contig length of ~3.1 Gb (contig N50 of 12.68 Mb, scaffold N50 of 141.27 Mb) and resolved two haplotypes. Structural variations were detected between the two haplotypes, among which two major inversions may be linked with ginger infertility. We further identified a relatively ancient whole-genome duplication (WGD) event within *Zingiberaceae*. By combining genomic data with RNA-seq data, we investigated allelic expression patterns and generated a gene coexpression network to better understand the spatiotemporally coordinated expression of alleles genome wide. We also annotated the allelic genes of the 6-gingerol biosynthesis pathway and predicted the transcription factors (TFs) that likely regulate 6-gingerol biosynthesis. The genome data and analyses reported here are of great scientific significance not only for understanding allele-specific gene expression but also for further functional research and breeding in ginger.

## Results

### Genome assembly

We sequenced a cultivated ginger individual with a diploid genome (2n = 2x = 22) whose chromosome number was verified by cytogenetic studies (Fig. S1). We generated ~330 Gb of ONT long reads, ~280 Gb of Hi-C paired-end reads, and ~145 Gb of Illumina PCR-free short reads (Table S1 and S2). The size of the diploid genome was estimated to be ~3.2 Gb using *K*-mer analysis (Fig. S2), corresponding to a haploid genome size of ~1.6 Gb, similar to the previously estimated size (~1.57 Gb)^12^. We compared multiple assembly strategies in the primary step, and based on contiguity metrics including N50, L50, and cumulative size, the “best” primary assembly (v0.3) was selected for further refinement and polishing (Table S3). Then, we obtained a genome assembly ready for Hi-C scaffolding, which was 3.09 Gb in length, including 1185 contigs with an N50 length of 5.74 Mb (Table S4).

**Fig. 1:**
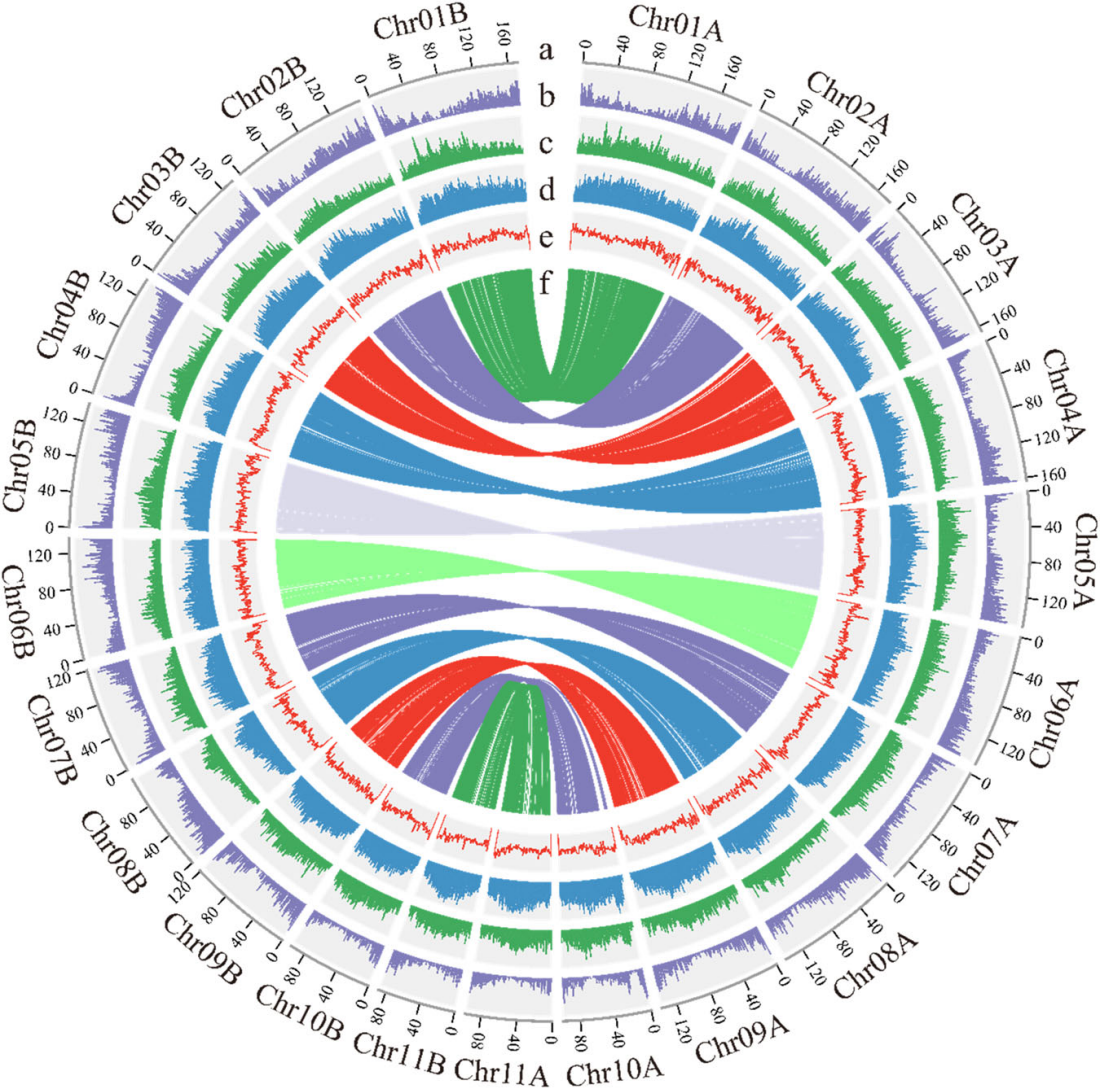
Overview of the haplotype-resolved genome assembly of cultivated ginger. The tracks (moving inwards) indicate the distribution of diverse genome features. **a** Length (Mb) of chromosomes. **b** Gene density. **c** *Gypsy* density. **d** *Copia* density. **e** Guanine-cytosine (GC) content. **f** Links between the core connected alleles. The identity of the longer haplotype of the pair of allelic chromosomes was affiliated with genome A, and that of the shorter haplotype was affiliated with genome B. All statistics were computed for windows of 1 MB

Next, with the Hi-C data, the final assembly was generated by anchoring 606 contigs to 22 superscaffolds (pseudochromosomes) with a total length of 3.05 Gb. Even without an assembler/setting specific to the phased assembly approach employed in our computational pipeline, we obtained a chromosome-scale haplotype-resolved assembly with 11 homologous chromosome pairs of the diploid ginger (Fig. 1, Table 1, Fig. S3; also see the following sections for the comparison of homologous chromosomes). Given the remarkable structural divergence between homologous chromosomes (haplotype genomes, shown in next sections), we attempted to separate the two sets of haplotype genomes by examining the potential bias in genome characteristics (such as the distribution of various TE families, guanine-cytosine (GC) contents, and *K*-mers). We failed, however, as no distinct patterns were found between any pair of allelic chromosomes. Because we also had no parental information on cultivated ginger, we arbitrarily denoted the longer chromosome from each pair of homologous chromosomes as coming from haplotype genome A and the other chromosome as coming from haplotype genome B (Fig. 1). The length of the pseudochromosomes ranged from 88.69 to 194.39 Mb (Table S5). After mapping the Illumina reads to the final assembly, SNPs were identified with SAMtools v1.8^13^, and we obtained a SNP heterozygosity of ~0.041% and a single-base error rate of ~0.0014%, suggesting that there were only a few regions with high sequence similarity (such as different haplotypes or repeat regions) that were not well resolved. Approximately 99.85% of the Illumina short reads could be successfully mapped to the genome assembly, and ~99.5% of the assembly was covered by at least 20X ONT long reads, indicating that the current assembly covered most unique genomic regions and was highly accurate. We used BUSCO to evaluate the quality of our genome assembly and found that 1296 (90.0%) of the 1440 universal single-copy genes in the Embryophyta lineage were included in our gene predictions^14^, among which 240 (16.7%) were denoted as single-copy genes and 1056 (73.3%) were duplicated genes because of the diploid nature of the current genome assembly. Among the remaining BUSCO genes, 30 (2.1%) had only fragmented matches, and 114 (7.9%) were entirely missing (Table S6).

**Table 1:** Ginger genome assembly statistics

Assembly feature	Number/Size
Assembly size (Gb)	3.1
No. contigs	796
Anchored size (Gb)	3.05
Max. contig length (Mb)	64.56
Min. contig length (kb)	5
Contig N50 length (Mb)	12.68
Contig L50 count	67
Contig N90 length (Mb)	2.3
Contig L90 count	298
Total number of scaffolds	219
Max. scaffold length (Mb)	194.39
Min. scaffold length (kb)	5
Scaffold N50 length (Mb)	141.27
Scaffold L50 count	10
Scaffold N90 length (Mb)	97.71
Scaffold L90 count	20
Gap number	577
GC content (%)	39.18
Gene number	78,923
Repeat content (%)	81.7

### Gene and repeat annotations

Our assembly contains 2.47 Gb (81.7%) of repetitive sequences (Table 1). We masked the repeat regions and proceeded to annotate the genome using a comprehensive strategy combining evidence-based and ab initio gene predictions. In total, 73,006 gene models were predicted for this diploid-resolved genome, with an average gene length of 5501.91 bp (Table 1). There were an average of 36,503 allelic genes per set of haploid genomes (number of allelic genes in haplotype genome A: 35,833; number of allelic genes in haplotype genome B: 35,395; number of allelic genes in scattered contigs: 1778). In addition, we identified 716 ribosomal RNAs (rRNAs), 3514 transfer RNAs (tRNAs), and 1687 additional unclassifiable small noncoding RNAs (ncRNAs) (Table S7). Among the predicted protein-coding genes, 98.04% could be annotated against multiple protein-related databases (Table S8, see “Materials and methods”).

Overall, we identified 3,750,198 repeat elements, among which long terminal repeat retrotransposons (LTR-RTs) were the most abundant, making up 56.6% of the genome, and *Copia* and *Gypsy* elements were particularly plentiful, accounting for 33.66% and 21.69% of the genomic content, respectively (Table S9). LTR-RTs were shown to have been gradually accumulating in the ginger genome over the past 5 million years (Fig. S4). Interestingly, we observed some subfamilies in which remarkable numbers of elements overlapped with coding genes, such as the Ale (~300 overlapping with genes), Angela (~500), and Ivana (>400) subfamilies from *Gypsy* and the Tekay subfamily (>500) from *Copia* (Figs. S5 and S6), indicating that different types of LTR-RT subfamilies may have different preferences for insertion sites and different functional implications in the ginger genome. By comparing ginger to other related plant species (*Musa acuminata, Daemonorops jenkinsiana, Oryza sativa, Phalaenopsis equestris, Dioscorea rotundata, Xerophyta viscosa, Zostera marina*, and *Vitis vinifera*) with respect to LTR-RT accumulation and removal rates, we found that ginger is characterized by high LTR-RT accumulation (*I* = 38.07) but low removal rates (*S*:*I* = 2.06), which could explain the overall higher proportion of transposable elements (TEs) in ginger and its larger genome size (the size of the haploid ginger genome is approximately 1.55 Gb) (Fig. S7).

### Comparative genomics and whole-genome duplication (WGD) events

**Fig. 2:**
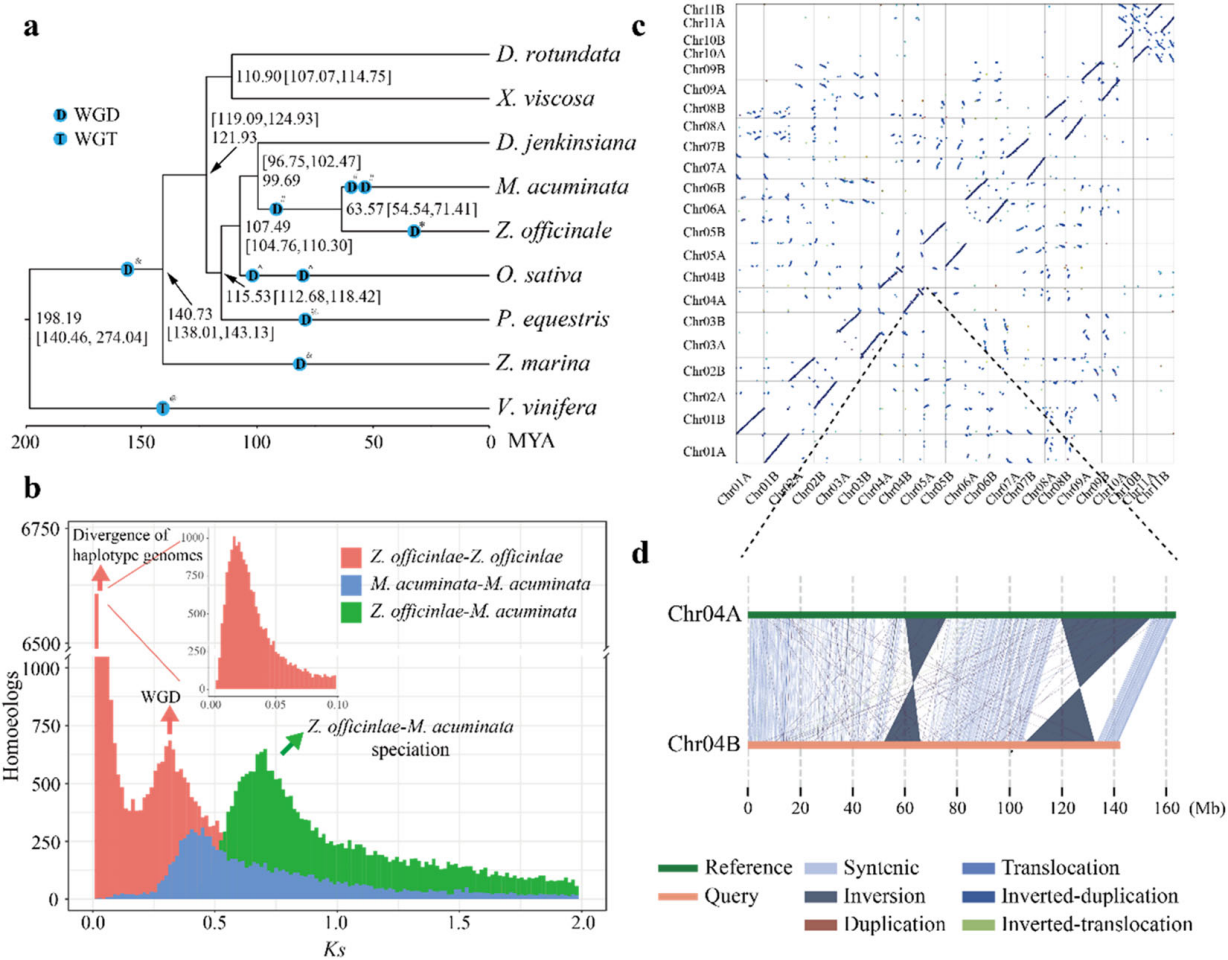
Genome evolutionary history. **a** Inferred phylogenetic tree, divergence times, and whole-genome duplications (WGDs). *WGD identified in this study; ^#^WGD reported in D’hont et al.^16^; ^%^WGD reported in Cai et al.^64^; ^^^WGD reported in Yu et al.^94^; ^&^WGD reported in Olsen et al.^78^; ^@^WGD reported in Jiao et al.^95^. **b** *Ks* distribution. *y* axis, *Z. officinlae* paralogs (red), *M. acuminata* paralogs (blue), *Z. officinlae*—*M. acuminata* orthologs (green). **c** Dot plots of paralogs in ginger. **d** Structural variations (SVs) between chromosomes 4A and 4B

To investigate the ginger genome evolution, we compared its genome to those of 7 other monocots, taking *V. vinifera* (dicotyledon) as an outgroup (Fig. 2a, see “Materials and methods”). We used OrthoFinder2^15^ to identify a total of 15,896 gene families consisting of 231,591 genes. A phylogenetic tree constructed from 163 single-copy orthologs confirmed the phylogenetic relationships of Zingiberales (*Z. officinale*), Arecales (*D. jenkinsiana*) and Poales (*O. sativa*) (Fig. 2a), which were in accordance with previous phylogenetic studies^16^. We identified 6590 gene families shared among these species, 227 species-specific families, and 1081 genes that did not cluster with orthologous clusters.

WGDs have played a major role in plant genome evolution^17^. The distribution of synonymous substitutions per site (*K*_*s*_) between syntenic gene pairs, synteny relationships, and inferred dating times were used to reveal WGD events and estimate duplication times. Our analysis indicated that the divergence time of ginger and *Musa* was approximately 63.57 MYA (*K*_*s*_ = 0.7) (Fig. 2b), while the *K*_*s*_ peaks suggestive of WGDs in both *Musa* and ginger were lower than 0.7 (~0.4 and ~0.3, respectively, Fig. 2b), indicating that the most recent WGD event (*K*_*s*_ = 0.3–0.4), estimated at ~27 MYA (*K*_*s*_ = 0.3), occurred after the divergence of *Musaceae* and *Zingiberaceae* (Fig. 2b, Figure S8, see “Materials and methods”). This *Zingiberaceae*-specific WGD event in the ginger genome was further supported by synteny analysis (Fig. 2c). In addition, we observed a weaker signal of at least one additional ancient WGD shared between ginger and *Musa*, which has been reported previously based on transcriptome data^16^ (Fig. S9).

### Structural variations between haplotype chromosomes

Genomic variation is a major contributor to genetic diversity and adaptive evolution and may be an important cause of speciation through recombination^18^. Structural variation refers to genomic alterations with a wide size range, including inversions, translocations, and duplications (or deletions). The high sterility of ginger pollen has been suggested to be a result of meiotic abnormalities caused by cytological factors such as heterozygosity for interchanges and heterozygous paracentric inversions^19–23^. Here, we identified a total of 46,902 structural variations (SVs) between the two haplotypes by using SyRI^24^ (Table S10). Among these SVs, we identified 281 inversions ranging in size from 882 bp to 35,629,541 bp, with a median size of 9.77 kb; 17,445 duplications ranging from 504 to 70,145 bp, with a median of 3301 bp; 16,787 inverted duplications ranging from 504 to 71,131 bp, with a median of 3244 bp; 6367 translocations ranging from 504 to 81,390 bp, with a median of 3921 bp; and 6022 inverted translocations ranging from 510 to 68,650 bp, with a median of 3,882 bp (Table S11). Among all SVs found in the ginger genome, the total length of inversions was longest, affecting 139 Mb of haplotype genome A (8.58%) and 114 Mb of haplotype genome B (7.97%) (Figs. S10, S11, and Table S11). It has been shown that structural chromosomal abnormalities are a key factor leading to infertility^25^. Reproductive failure would be expected to occur because of the production of chromosomally unbalanced gametes following abnormal meiotic events^26^. In ginger, two large inversions (28–36 and 15-16 Mb), were found on chromosome 4 (Fig. 2c, d, Fig. S11), which may be the key factors inducing the observed meiotic abnormalities leading to the infertility of cultivated ginger.

### Spatiotemporal expression pattern of alleles

**Fig. 3:**
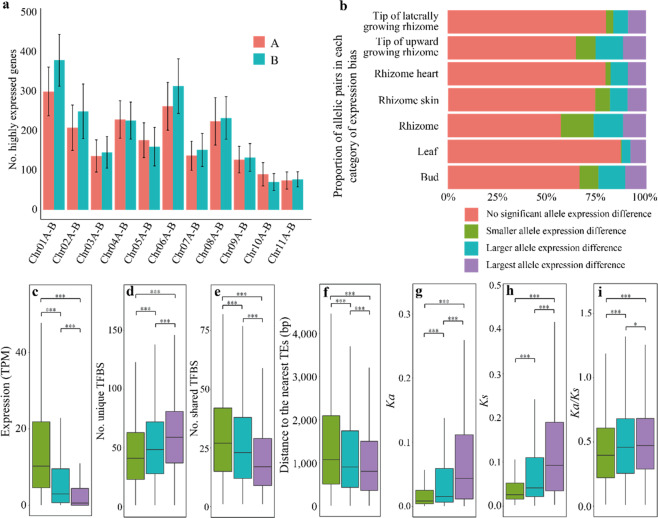
Allelic gene expression among haplotype chromosomes. **a** Numbers of highly expressed genes on haplotype chromosomes in seven different tissues. Graphs display the average numbers of highly expressed genes ± s.d. **b** Proportions of alleles in each category of allelic expression bias across the seven individual tissues and global tissues. **c** Absolute TPM expression abundance for the smaller allele expression differences, larger allele expression differences, and largest allele expression differences. **d** Numbers of specific transcription factor binding sites (TFBSs) for the smaller allele expression differences, larger allele expression differences, and largest allele expression differences. **e** Numbers of shared TFBSs for the smaller allele expression differences, larger allele expression differences, and largest allele expression differences. **f** Distances to the nearest TEs for the smaller allele expression differences, larger allele expression differences, and largest allele expression differences. **g**–**i** *K*_*a*_, *K*_*s*_, and *K*_*a*_/*K*_*s*_ ratios for the smaller allele expression differences, larger allele expression differences and largest allele expression differences. The data for (**c**–**i**) are presented in box-and-whisker plots. The bottom and top of each box represent the 25th and 75th percentiles, respectively. The center line represents the 50th percentile. The whiskers indicate the minimum and maximum values. Mann–Whitney–Wilcoxon test. *p < 0.05; **p < 0.01; ***p < 0.001. **c**–**i** Colors are consistent with cluster colors in (**b**)

In diploid plants, many quantitative trait variations are regulated by genetic interactions between alleles^27^. These interactions range from buffering effects observed during functional redundancy to the mutation of a single allele that can lead to a dominant effect of a phenotype. Understanding allelic expression profiles will aid in developing strategies for improving crops by locating and manipulating single or multiple alleles to quantitatively regulate trait responses, especially as versatile precision genome editing is now being established and widely applied to crop breeding^28^. Using MCscanX^29^, we identified 43,438 allelic genes at 21,719 loci between the allelic chromosome pairs in the haplotype-resolved ginger genome (Fig. 1, Fig. S12, see “Materials and methods”). In global surveys across seven different tissues (leaves, buds, rhizomes, rhizome hearts, rhizome skin, tips of laterally growing rhizomes, and tips of upward growing rhizomes, Table S1), although 4922 (22.7%) alleles showed an over 2-fold difference in expression, most alleles did not exhibit an over two-fold difference in expression, suggesting that most alleles were coordinately expressed in the ginger genome. The number of highly expressed alleles (with an over 2-fold difference in expression) showed no significant difference between allelic chromosome pairs (Fig. 3a), suggesting that expression was generally not biased between the two haplotypes.

Based on the different expression patterns observed across seven different tissues, we defined the following allelic expression bias categories: (1) no significant allele expression difference (*p* ≥ 0.05); (2) smaller allele expression differences with a fold-change (FC) ≤ |2| (*p* < 0.05); (3) larger allele expression differences with a |2| < FC < |8| (*p* < 0.05); and (4) largest allele expression differences with FC ≥ |8| (*p* < 0.05).

Most alleles showed no significant differential expression within the same tissue (no significant allele expression difference; 55–87% of alleles, Fig. 3b). The alleles showing the largest allele expression differences were relatively stable in different tissues, accounting for 7–12% of the alleles within the different tissues (Fig. 3b). The alleles from the smaller allele expression difference category showed higher absolute transcriptional abundance than the alleles in the larger allele expression difference and largest allele expression difference categories (Fig. 3c), indicating that allelic expression was not the result of an overall increase in the expression of a single allele but were instead the result of a relative decrease in the expression of an allele. In addition, we found that the differences in expression were tightly associated with the difference in the number of shared and unique transcription factor binding sites (TFBSs) between the two alleles. Indeed, the alleles of the smaller allele expression difference group had more shared TFBSs, and those of the largest allele expression difference group had more unique TFBSs (Fig. 3d, e). We further examined the associations of flanking TEs with the expression difference between alleles and found a significant association of TE proximity with differences in allelic expression. Alleles in the largest allele expression difference group were found to be significantly closer to the TEs (Fig. 3f).

To determine whether selection pressure is related to differences in allelic expression, we compared the rates of nonsynonymous mutations (*K*_*a*_), synonymous mutations (*K*_*s*_), and the ratio of *K*_*a*_/*K*_*s*_ between the alleles at each allelic locus. We observed that the alleles from the largest allele expression difference category showed significantly higher *K*_*a*_, *K*_*s*_, and *K*_*a*_/*K*_*s*_ values than the alleles from other categories (Fig. 3g–i), suggesting that these alleles experienced sequence divergence, which may underlie the differences in allelic expression.

**Fig. 4:**
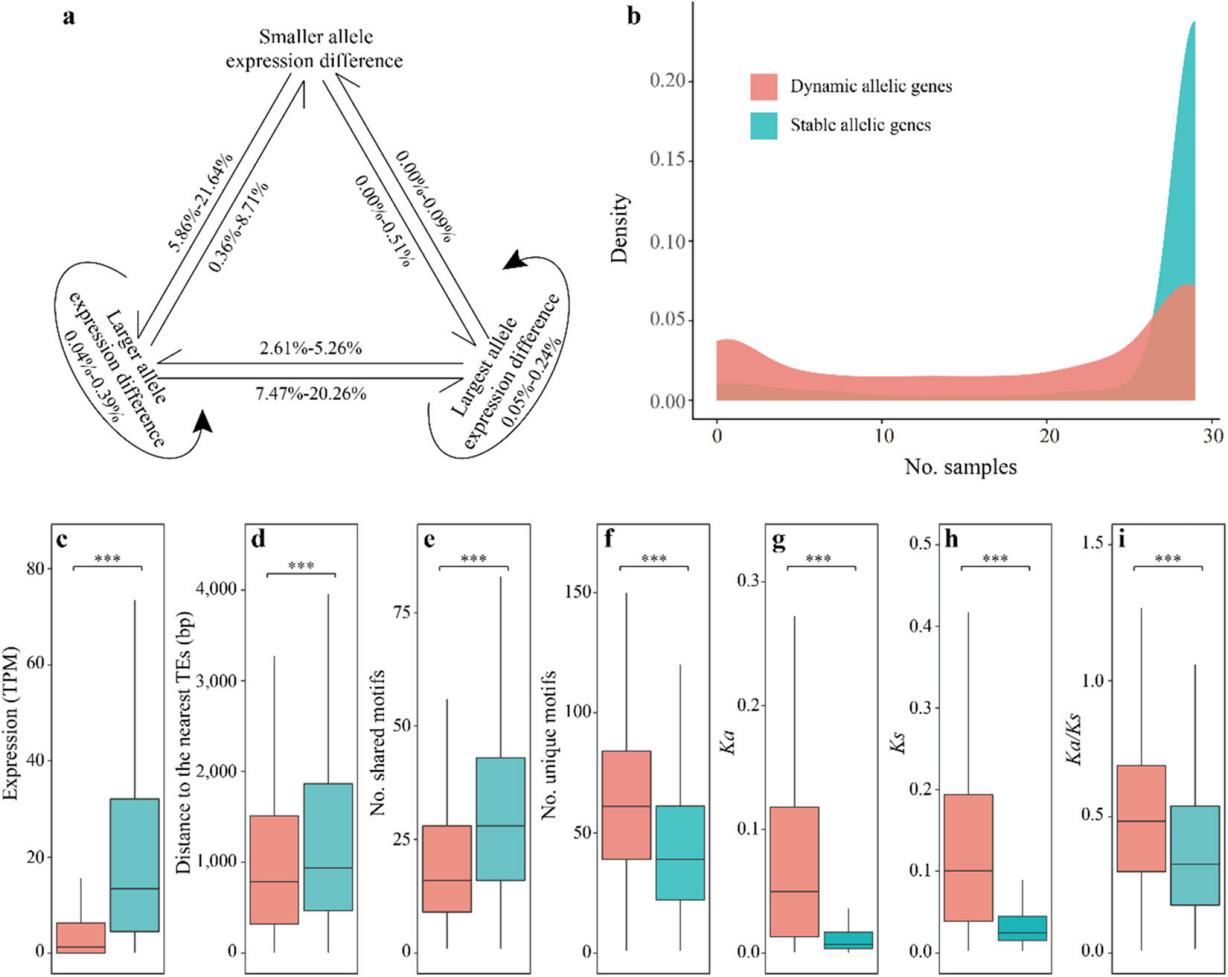
Dynamic and stable allelic expression patterns. **a** Variation in smaller allele expression differences, larger allele expression differences, and largest allele expression differences. **b** Numbers of samples in which stable and dynamic alleles are expressed. **c** Absolute TPM expression abundance for dynamic and stable alleles. **d** Distance to the nearest TEs for dynamic and stable alleles. **e** Numbers of shared transcription factor binding sites (TFBSs) for dynamic and stable alleles. **f** Numbers of specific TFBSs for dynamic and stable alleles. **g**–**i** *K*_*a*_*, K*_*s*_, and *K*_*a*_*/K*_*s*_ ratios for dynamic and stable alleles. The data for (**c**–**i**) are presented in box-and-whisker plots. The bottom and top of each box represent the 25th and 75th percentiles, respectively. The center line represents the 50th percentile. The whiskers indicate the minimum and maximum values. Mann–Whitney–Wilcoxon test. *p < 0.05, **p < 0.01; ***p < 0.001. Colors are consistent with the cluster colors in (**b**)

Our above analyses provide information about the static expression of alleles in different tissues. Therefore, we explored whether alleles retained their biased expression among different tissues. We found that most genes retained their allelic expression biases, with only 0.00–21.64% (0–595) changing in at least one different tissue (Fig. 4a, Table S12). Approximately 0.36–21.64% (10–595) of alleles changed between neighboring categories (i.e., category change from smaller allele expression difference to larger allele expression difference, larger allele expression difference to largest allele expression difference), while only 0.00–0.51% (0–14) alleles jumped over categories (i.e., category change from a smaller allele expression difference to the largest allele expression difference) (Fig. 4a, Table S12). These data showed that alleles were usually stably expressed in different tissues.

Based on the dynamic changes in the expression differences between different tissues (see “Materials and methods”), we focused on the 10% most “stable” alleles, showing consistent expression among tissues, and the 10% most “dynamic” alleles, showing significantly different expression among tissues. Stable alleles were highly expressed relative to dynamic alleles and showed higher expression breadths/values, being expressed across almost all samples, whereas dynamic alleles showed more tissue-specific expression (Fig. 4b, c). Stable alleles were enriched for functions related to mRNA polyadenylation, mRNA export from the nucleus, and tRNA methylation (Table S13), whereas dynamic alleles were enriched for defense-related functions, such as terpenoid biosynthetic processes, monoterpene biosynthetic processes, and sesquiterpene biosynthetic processes (Table S14). Compared to the stable alleles, the dynamically expressed alleles had significant flanking TEs in their proximity (Fig. 4d), suggesting that flanking TEs contribute more to the variation in the relative expression of alleles. In addition, the dynamic alleles had fewer conserved TFBS motifs and significantly higher *K*_*a*_, *K*_*s*_, and *K*_*a*_/*K*_*s*_ values than the stable alleles (Fig. 4e–i), suggesting that the dynamic alleles according to spatial expression patterns were under more relaxed selection and showed more frequent mutations.

These results indicated that differences in spatiotemporal expression patterns were positively related to the proximity of flanking TEs, differences in TFBS, selection pressure, and sequence mutations. Differences in spatiotemporal expression patterns and relaxation of selection may also lead to functional innovation, potentially related to the wide adaptability of ginger.

To better understand the spatiotemporally coordinated expression between alleles, we performed a coexpression network analysis incorporating all expressed alleles. We found that 49.14% of alleles were in the same coexpression module, suggesting highly coordinated expression patterns (Table S15). To quantify the “similar” or “divergent” expression patterns of the alleles outside the module (50.86%), we calculated a threshold based on the pairwise distance of coexpression between alleles (see “Materials and methods”). We found that 29.63% of the alleles had a divergent pattern, suggesting that only a few alleles showed divergent expression and that most alleles were actually expressed in coordination (Table S15). GO enrichment analysis revealed that these divergent alleles were mainly related to resistance, showing enrichment in categories such as response to toxic substance, terpenoid biosynthetic process, and alkaloid metabolic process (Table S16).

### 6-Gingerol biosynthesis and its genetic regulation network

**Fig. 5:**
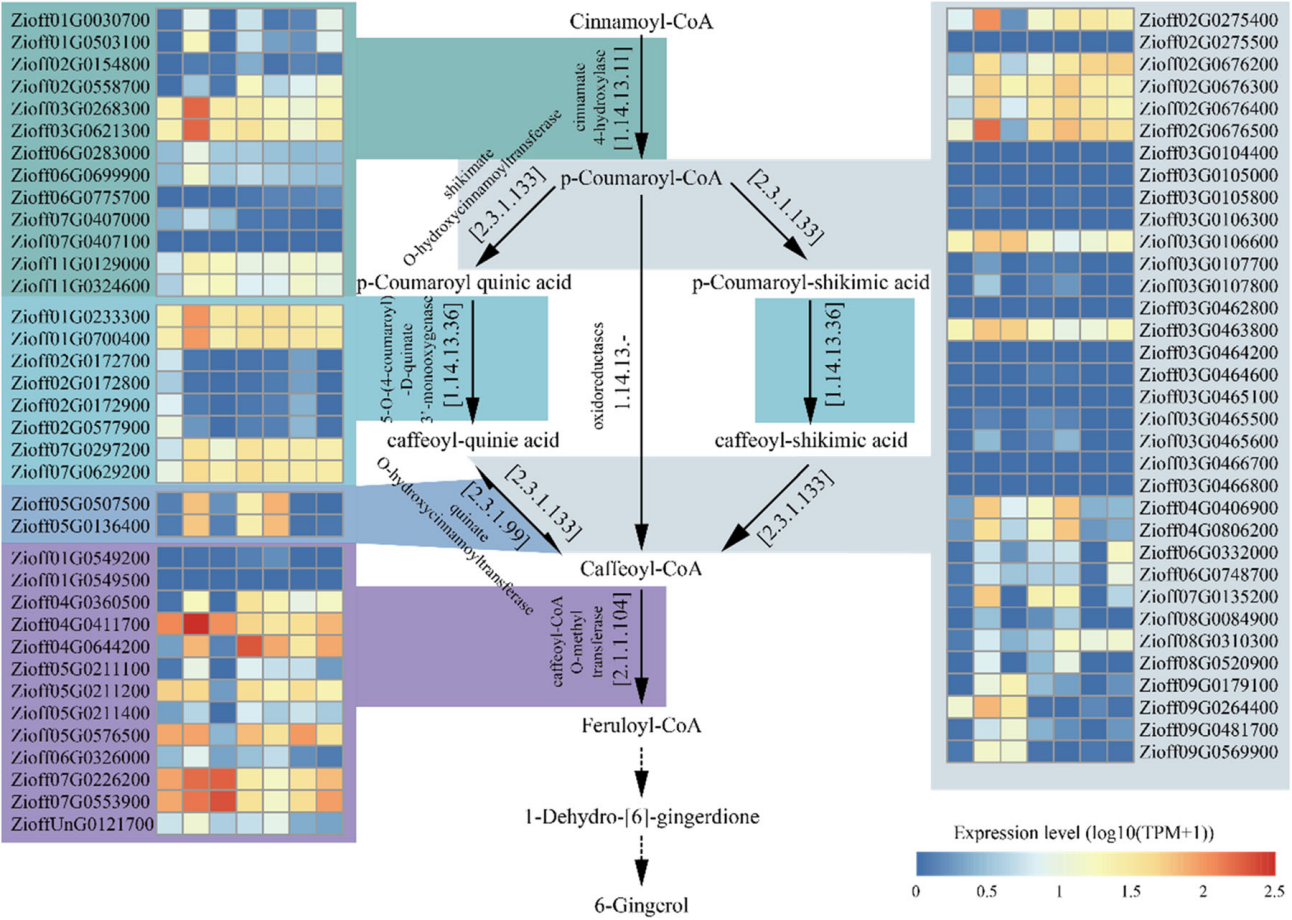
The biosynthesis pathway of 6-gingerol. The gene expression profiles (log_10_(TPM + 1)) of different tissues (rhizome, leaf, bud, tip of laterally growing rhizome, tip of upward growing rhizome, rhizome heart, rhizome skin; from left to right in each heatmap panel) are presented in the heatmap alongside the gene names

6-Gingerol is the main ingredient of ginger essential oil, which provides the unique flavor and medicinal value of ginger. In the 6-gingerol biosynthesis pathway, cinnamoyl-CoA is first converted to p-coumaroyl-CoA by cinnamate 4-hydroxylase (EC:1.14.13.11). Next, p-coumaroyl-CoA synthesizes caffeoyl-CoA through three pathways and is then converted to feruloyl-CoA by caffeoyl-CoA O-methyl transferase (EC:2.1.1.104). Finally, feruloyl-CoA is converted to 1-dehydro-[6]-gingerdione and then to 6-gingerol by uncharacterized enzymes (Fig. 5). In the ginger genome, a total of 70 allelic genes were mapped to the 6-gingerol biosynthesis pathway (Fig. 5).

The shikimate O-hydroxycinnamoyltransferase enzyme (EC: 2.3.1.133), a type of acyltransferase, may be a rate-limiting enzyme in the 6-gingerol biosynthesis pathway^30^ and thus play an important role in regulating the biosynthesis of 6-gingerol. Several allelic genes (*Zioff02G0275400*, *Zioff02G0676200*, *Zioff02G0676300*, *Zioff02G0676400*, *Zioff02G0676500*, *Zioff03G0106600*, *Zioff03G0463800*) encoding shikimate O-hydroxycinnamoyltransferase enzymes were highly expressed in all rhizome-related tissues (Fig. 5), suggesting that these allelic genes may represent the key steps in the 6-gingerol biosynthesis pathway (Figure S13). These enzyme-encoding genes originated from an ancient common WGD characterized in all monocots (Figure S13). The subsequent occurrence of tandem repeats caused the genes encoding the enzyme to expand rapidly.

**Fig. 6:**
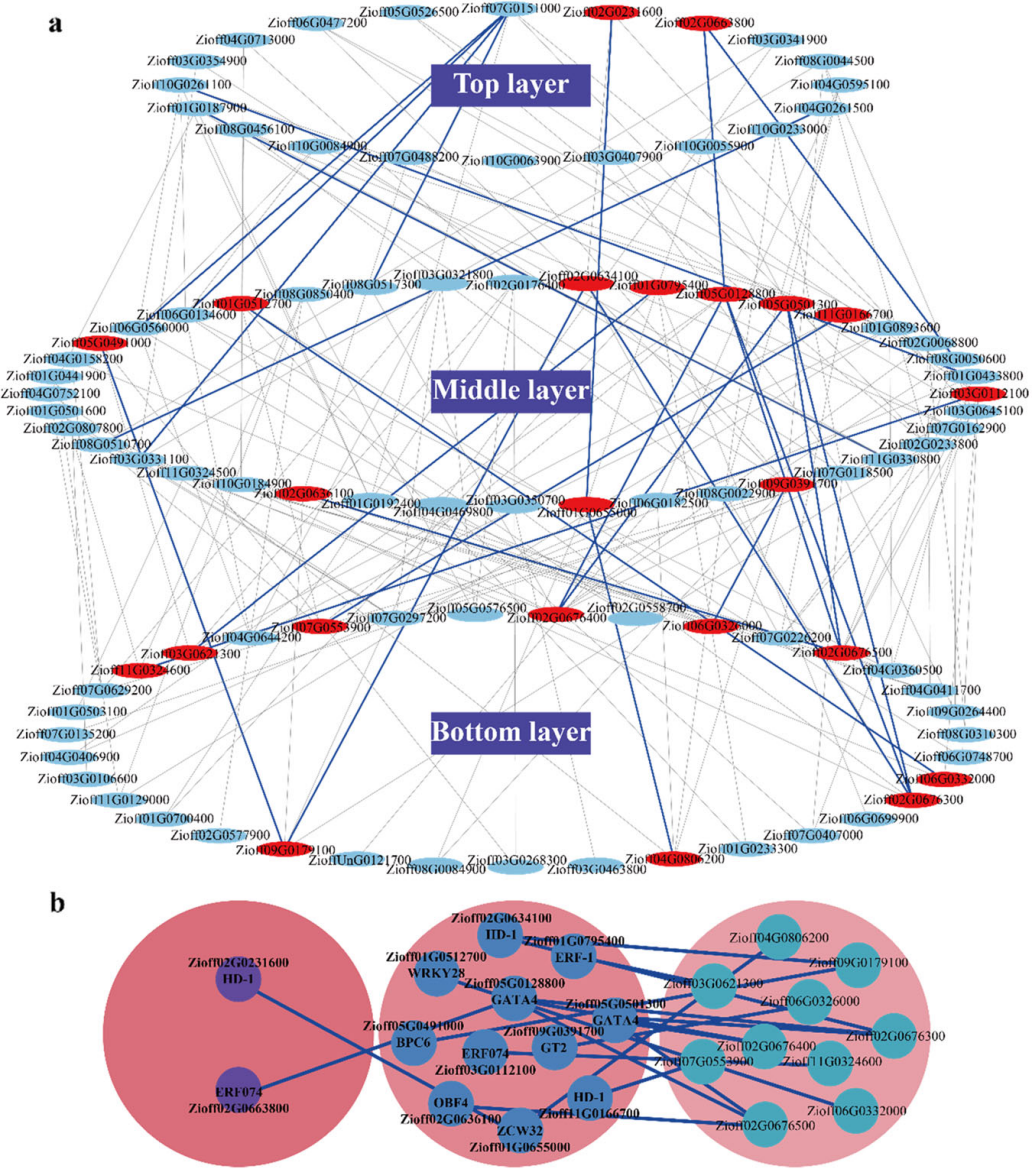
Gene regulatory network (GRN) related to 6-gingerol biosynthesis identified with the BWERF algorithm. **a** Multilayer hierarchical GRN related to 6-gingerol biosynthesis identified with the BWERF algorithm. Gray and blue lines represent regulatory roles; blue lines represent regulatory roles also supported by TFBS predictions. Red nodes in the top and middle layers represent key TFs that hierarchically regulate enzyme-coding genes (red nodes in the bottom layer) of the 6-gingerol biosynthesis pathway. **b** Subnetwork of key transcription factors (TFs) and their regulatory pathway genes

To explore the key factors involved in regulating 6-gingerol biosynthesis, we constructed a multilayer hierarchical gene regulatory network governing the 6-gingerol biosynthesis pathway. As a result, 35, 40, and 20 allelic genes were found to be located in the bottom, middle, and top layers, respectively (importance score >6.5; Fig. 6a). Sixty TFs were found to potentially regulate 35 downstream 6-gingerol biosynthesis pathway genes through this hierarchical network. Furthermore, from this multilayer hierarchical gene regulatory network and TFBS prediction, we identified 2 key TFs (*HD-ZIP-1*, *Zioff02G0231600*; *ERF074*, *Zioff02G0663800*) in the top layer that may potentially target TFBSs in the promoter regions of 2 other TFs (*ZCW32*, *Zioff01G0655000*; *GATA4*, *Zioff05G0128800*) in the middle layer, and these 2 TFs could further target TFBSs in the promoter regions of four enzyme-coding genes in the bottom layer of the 6-gingerol biosynthesis pathway (Fig. 6a, b, Table S17). Additionally, 9 key TFs (*OBF4*, *Zioff02G0636100*; *BPC6*, *Zioff05G0491000*; *WRKY28*, *Zioff01G0512700*; *HD-ZIP-1*, *Zioff02G0634100*; *ERF-1*, *Zioff01G0795400*; *GATA4*, *Zioff05G0501300*; *HD-ZIP-1*, *Zioff11G0166700*; *ERF074*, *Zioff03G0112100*; *GT2*, *Zioff09G0391700*) in the middle layer could target TFBSs in the promoter regions of enzyme-encoding genes in the bottom layer of the 6-gingerol biosynthesis pathway (Fig. 6a, b, Table S17). These key TFs probably play critical roles in regulating the biosynthesis of 6-gingerol and will be targets of further functional verification.

## Discussion

Here, we present a high-quality chromosomal-scale phased genome assembly for the key spice and medicinal species ginger from two haploids. We identified one independent WGD event unique to *Zingiberaceae*. Abundant structural variations were identified between the two haplotypes. In particular, two large inversions were observed on chromosome 4, in line with previous cytological studies that have identified heterozygous paracentric inversions, and these inversions are likely responsible for subsequent meiotic abnormalities leading to pollen sterility^19–23^ and infertility in cultivated ginger^7^ and further support the hybrid origin of cultivated ginger.

Our results showed that most alleles were expressed in coordination and that only a small number of alleles showed >2-fold changes in expression, and our coexpression network analysis further verified these results. However, differential gene expression was still observed between alleles, potentially related to the wide adaptability of ginger. Sequence differences, TE proximity, selective pressure, and TFBSs may be important reasons for changes in allelic expression. TEs are usually associated with gene expression^31^. These mobile genetic elements may be responsible for the initial differences in gene expression and further promote subfunctionalization. A relaxed level of purification selection causes newly inserted TEs located near genes to be removed less efficiently, resulting in more adjacent silent TEs^32^. Therefore, TEs may be a cause of allelic differentiation, a result of allelic differentiation, or both.

Plant secondary metabolism plays an important role in development and ecology, for example, by producing defense agents and signals, and is widely exploited in medicine and the production of dyes and condiments^33^. The pungency of ginger is conferred by 6-gingerol. The fresh aroma and pungent taste of ginger have made it an essential ingredient in many dishes and the food processing industry around the world. Based on the high-quality genome, we annotated the genes of the 6-gingerol biosynthetic pathway. Furthermore, for these genes, we constructed a multilayered hierarchical gene regulatory network. Several important TFs have been identified as key elements in coordinating the biosynthesis of 6-gingerol. These results provide a valuable reference for further functional studies and molecular breeding in ginger.

In conclusion, the high-quality haplotype-resolved ginger genome and transcriptional landscape reported herein provide a key reference and framework for the further development of genome editing in plant biotechnology. The high-quality genome will be helpful for molecular breeding and genetic, genomic, and evolutionary studies in both ginger and related species.

## Materials and methods

### Plant material

“Zhangliang” ginger is a ginger landrace passed down from the Chinese Han dynasty that has been propagated for more than 2000 years through asexual reproduction. This variety is now endemic to Lushan County, Pingdingshan, Henan Province, China, and is well known for its strong pungent flavor and richness in gingerol, ginger oil, and amino acids. The sequenced “Zhangliang” individual was collected from Zhangliang Town, Lushan County, Pingdingshan, Henan, China. The rhizomes were harvested and immediately frozen in liquid nitrogen before genomic DNA extraction.

### Determination of chromosome number and ploidy

The diploid nature of the sequenced individual was determined (2n = 2x = 22) using a classical cytological method. Briefly, rhizomes were collected and pretreated in a saturated solution of paradichlorobenzene for at least 3 h, washed once using distilled water, and then fixed in Carnoy’s fluid (ethanol/acetic acid, 3:1) for at least 24 h at 4 °C. Next, the materials were transferred to 1 mol/L HCl for 10–15 min at 60 °C, washed with water, and then immersed in distilled water for 10 min. These hydrolyzed materials were stained with carbolfuchsin solution. The chromosome counts of at least 10 cells per rhizome with well-spread metaphases were observed using a microscope (Olympus CX23; Olympus, Tokyo, Japan).

### Genome sequencing with ONT and Illumina technologies

We used the phenol-chloroform and CTAB methods^34^ to extract high-quality genomic DNA from rhizomes for ONT and Illumina sequencing. For ONT sequencing, PromethION libraries were prepared following the Oxford Nanopore 1D genomic DNA by ligation (SQK-LSK109) – PromethION (version GDE_9063_v109_revD_04Jun2018) protocol and sequenced on the Nanopore PromethION platform. For Illumina sequencing, following the original Illumina TruSeq DNA PCR-free LPP (revision A, January 2013, low sample with 550 bp insert size) protocol, PCR-free library preparation was also performed, and the library was then sequenced with an Illumina HiSeq X Ten platform.

### RNA extraction and library preparation

We harvested clones of the same individual used for whole-genome sequencing. Tissues including leaves, buds, rhizomes, rhizome hearts, rhizome skin, tips of laterally growing rhizomes, and tips of upward growing rhizomes were sampled and treated with liquid nitrogen. Total RNA was extracted with TRIzol reagent, and mRNAs were purified using a NEBNext Ultra RNA Library Prep Kit for Illumina sequencing (New England Biolabs Inc.). Two micrograms of RNA from each sample was used to prepare the RNA-seq libraries and then sequenced on the Illumina HiSeq X Ten platform.

### Hi-C library preparation and sequencing

We followed a standard procedure to prepare the Hi-C library^35^. In brief, in situ cross-linked DNA was extracted from 700 ng of high molecular-weight genomic DNA and digested with a restriction enzyme. The sticky ends of the digested fragments were biotinylated, diluted, and randomly joined to form chimeric junctions. Biotinylated DNA fragments were enriched and sheared to a size of 300–500 bp in the step of sequencing library preparation, and sequencing was then performed on the Illumina HiSeq X Ten platform.

### Genome size estimation through *K*-mer analysis

The genome size of ginger was estimated using *K*-mer analysis. Briefly, *K*-mer counting was conducted using JELLYFISH^36^. Genome size was estimated with GCE v1.0.0^37^. Specifically, genome size = (total *K*-mer)/(position of homozygous peak).

### Genome assembly

The SMARTdenovo v1.0^38^ and wtdbg2 v2.1^39^ assemblers with ONT reads corrected by Canu v1.7^40^ were used for de novo assembly. The following settings were used in SMARTdenovo: -c 1 to generate a consensus sequence, -J 5000 to remove sequences <5 kb and -k 19 to use 19-mers, as advised by the developers for large genomes. Based on contiguity metrics including N50, L50 and cumulative size, the ‘best’ assembly (v0.3) was selected for further refinement and scaffolding. Since ONT reads contain systematic errors in homopolymeric regions, we mapped Illumina reads to the assembled genome using BWA v0.7.15^41^ and then conducted polishing seven times by using Pilon v1.22^42^ to enhance the single-base accuracy.

### Scaffolding with Hi-C data

We mapped the clean Hi-C reads to the assembly with Juicer^43^ and used the 3d-DNA v180922 pipeline^44^ to correct misjoins, order, and orientation. Then, using Juicebox v1.8^43^, the assembly was manually reviewed and refined for quality control and interactive correction. Finally, we rescaffolded each chromosome with 3d-DNA separately to decrease the influence of chromosome interactions and improve the chromosome-scale assembly. Due to high heterozygosity between haplotypes, there were enough differences to allow us to obtain haplotype-resolved genomes.

### Optimization of the genome assembly

Using LR_Gapcloser v1.1^45^, the genome assembly was gap-closed twice. We further conducted five rounds of assembly polishing with NextPolish v1.1.0^46^ to improve base accuracy. Contigs with an identity higher than 98%, overlap greater than 80%, and lengths less than 5 kb were removed. BUSCO was used to assess genome completeness. Furthermore, ONT sequencing reads and Illumina reads were mapped to the genome assembly using BWA and Minimap2 v 2.11-r797^47^, respectively. SNP calling was executed with SAMtools v1.8 (default settings) to calculate SNP heterozygosity and the single-base error rate.

### Transcriptome assembly

RNA-seq reads were preprocessed with fastp v0.19.3^48^ to remove adapters and low-base-quality sequences. We mapped the RNA-seq reads to the genome with HISAT2 v2.1.0 and used StringTie v1.3.3b^49^ and Trinity v2.0.6^49^ for reference-guided assembly. We also used Trinity^50^ for de novo assembly. To achieve a more complete assembly, we also downloaded and incorporated ginger expressed sequence tags (ESTs) from NCBI. The assembled transcripts were combined using CD-HIT v4.6^51^.

### Characterization of repetitive sequences

Repeat families were first identified de novo and classified initially using RepeatModeler v1.0.10 (http://www.repeatmasker.org/RepeatModeler/). The repeat library produced by RepeatModeler was analyzed with RepeatMasker v4.0.7 (http://www.repeatmasker.org/) to further reveal repeats. LTRharvest^52^ and LTRdigest^53^ were used for the de novo prediction of LTR-RTs.

### Full-length LTR-RT annotation

We annotated the full-length LTR-TRs and further identified differences in the proliferation, age dynamics, and gene proximity of different LTR-RT families following a previously published reference^54^ with some modification. In brief, the LTR-RT candidates that possessed complete Gap-Pol protein sequences were retained as intact LTR-RTs (*I*), while solo-LTRs (*S*) and truncated LTRs (*T*) were identified based on sequence similarity to the intact LTR-TRs (E-value < 1e−10, overlap length >90%, identity >90%). Then, LTR homology both up- and downstream (15 kb) was extracted and compared with Gap-Pol protein sequences within the rexdb database^55^. The corresponding LTR-RTs were considered truncated LTR-RTs when they showed at least 50% Gag-Pol coverage by one side of the flanking sequence and 30% identity (E-value < 1e−8). The identified LTR-RTs lacking Gag-Pol up- and downstream of the LTRs were considered to be solo-LTRs.

### Differential proliferation, age dynamics, and gene proximity of different LTR-RT families

The insertion time of an LTR-RT was estimated according to the difference between the 5′-LTR and the 3′-LTR of the same transposon^56^ using MAFFT v7.221^57^ with a mutation rate of 1.3e−8 substitutions per year per site. Although the actual pattern of LTR-RT activation and amplification appeared at the family level, as defined by >80% sequence homology in the LTR-RTs, our focus was on holistic genomic characteristics that can be more carefully compared at the LTR-RT superfamily level (>60% homology). We calculated the distances between intact LTR-RTs and adjacent genes and examined the relationships of gene proximity and insertion times.

We aligned the 5′-LTR sequences of all LTR-RTs to understand the relationships among individual LTR-RTs. If two LTRs covered at least 70% of each others’ lengths and shared at least 60% identity, they were assigned to the same cluster^58^. We also compared Solo-LTRs and truncated LTR-RTs to the same cluster containing the 5′-LTRs from the most similar intact LRT-RTs. The ratios of the solo LTR-RTs and truncated LTR-RTs to intact LTR-RTs (*S*:*I*, *T*:*I*) and the sum thereof were then evaluated separately to investigate the removal rate of LTR-RTs over the past several million years. Furthermore, we evaluated the proportion of clusters with *S*:*I* values greater than 3.

### Gene annotation

After repetitive sequence masking, we used the Augustus^59^ ab initio gene finder to identify gene models. BLASTn and tBLASTx from BLAST v2.2.28+ ^60^ were used to map the transcriptome assembly to the genome; BLASTx was used to map protein sequences to the genome (protein models from 5 species: *Musa acuminate*^61^, *Oryza sativa*^62^, *Elaeis guineensis*^63^, *Phalaenopsis equestris*^64^, *Ananas comosus*^65^) for further optimization. The results were integrated to predict the gene model using Augustus. Finally, predicted gene models with abnormal frames (no start or stop codon or an inside stop codon) were excluded. tRNAScan-SE v1.3.1^66^ and RNAmmer v1.2^67^ were used to identify tRNAs and rRNAs, and other types of ncRNAs were identified by searching against the Rfam v9.1 database^68^. Predicted gene models were aligned to proteins in SwissProt^69^, TrEMBL^69^, the NCBI nonredundant protein database (NR), and Pfam^70^ and eggnog databases using blat v36^71^ (E < 1e5) to determine the best-matching alignments (identity > 30%). Using InterProScan v5.27-66.0^72^, motifs and functional domains were identified by searching against protein databases, including ProDom, PROSITE, Pfam, SMART, PANTHER, and PROSITE. In addition, we mapped the predicted genes to GO classification and KEGG pathways.

### Structural variation detection

The Nucmer alignment tool from the MUMmer v4.0.0 toolbox^73,74^ was used to perform whole-genome alignments. Nucmer was run with -maxmatch to obtain all alignments between two allelic chromosome pairs with the parameters -c 500, -b 500, and -l 100. The delta-filter and show-coords subprograms were employed to filter the alignments and convert them to tab-delimited files. Finally, SyRI^24^ was used to identify inversions, translocations, duplications, inverted translocations, and inverted duplications.

### Phylogenetic tree construction, divergence time estimation, gene family identification, and WGD analysis

The ginger proteome was globally compared with *M. acuminata*^16^, *D. jenkinsiana*^75^, *O. sativa*^62^, *X. viscosa*^76^, *P. equestris*^64^, *D. rotundata*^77^, *Z. marina*^78^, and *V. vinifera*^79^ proteomes filtered for alternative splicing. Orthofinder2 v2.3.1^15^ was used to identify homologous gene clusters. Based on 163 single-copy orthologs, we used IQ-TREE v1.6.7^80^ to construct a maximum likelihood (ML) phylogenetic tree with the best-fit model (JTT + F + R4). These single-copy protein sequences were further processed using MAFFT v7.427^57^ alignment and trimAL v1.4.rev22^81^ trim (-gt 0.8 -st 0.001 -cons 60) and were then converted to amino acid sequences. The ML phylogenetic tree and converted amino acid sequences were employed to estimate divergence times with MCMCtree of the PAML v4.9h^82^ using the approximate likelihood method with the independent substitution rate, HKY85 substitution model, 2.1e6 iterations and 1e5 iterations discarded as burn-in, and 3 fossil dating points from TimeTree (http://timetree.org) were taken as input: the 115–308 MYA split time between *V. vinifera* and *Z. marina*, the 125–141 MYA split time between *Z. marina* and *X. viscosa*, and the 100–118 MYA split time between *D. jenkinsiana* and *M. acuminata*. We checked the convergence by running the procedure in duplicate with results compared between runs. MCScanX^29^ was used for collinear analysis with default settings. The *K*_*a*_ and *K*_*s*_ values of alleles/gene pairs were calculated by using KaKs_Calculator v2.0^83^ with the Yang-Nielsen (YN) model. We excluded *K*_*s*_ values >5.0 from all analyses due to saturated substitution as synonymous sites^84,85^. The *K*_*s*_ values of *Musa*–ginger orthologs with the speciation dating of the two species allowed the calculation of the number of substitutions per synonymous site per year (divergence date = *K*_*s*_/(2 * r)). The same r value was applied to ginger WGD/divergence events and their peak *K*_*s*_ values to calculate WGD ages.

### Allele identification

MCScanX^29^ was used to identify collinear block gene pairs between a pair of allelic chromosomes with default settings. Then, we manually removed collinear blocks that probably resulted from WGD. Finally, we verified the accuracy of the identified alleles through visualization.

### Gene expression

The RNA-seq clean reads of each sample were aligned to the ginger genome using HISAT2 v2.0.0^86^. The normalized TPM values of each sample were estimated with featureCounts v1.5.3^87^. We established the following standard: if the gene/allele expression value of at least 1 sample among the 29 given samples exceeded 0.5 TPM, we considered the gene/allele to be expressed. The DESeq2 package^88^ was used to investigate differences in expression between alleles. The following FC value ranges were used as criteria for selecting differentially expressed alleles: 8 ≥ FC ≥ 2 or FC > 8 or 2 > FC ≥ 0 with an adjusted *p* < 0.05.

### Dynamic and stable allele identification

We first identified alleles with an FC ≥ 2 (*p* < 0.05) in each tissue. If the *p* value was greater than 0.05 in one tissue, we considered the pair of alleles to show no difference (FC = 0) in expression. Dynamically expressed alleles were defined as the top 10% of alleles with the largest FC change across seven different tissues, and stably expressed alleles were defined as the top 10% of alleles with the smallest FC change.

### Transcription factor (TF) and transcription factor binding site (TFBS) identification

The protein sequences of all annotated genes were submitted to plantTFDB^89^ to identify the TFs with the best hits to TFs of *Arabidopsis thaliana*. The 2 kb sequences upstream of the genes were used to identify TFBSs present in the promoters of genes. MEME v4.12^90^ was used with a position weight matrix (PWM) obtained from plantTFDB^89^ to predict TFBSs based on a set of manually curated, nonredundant, and high-quality TF binding motifs derived from experiments (*p* < 1e−05, -motif-pseudo of 1e−08 and a -max-stored-scores of 1e6).

### Gene coexpression network

We selected expressed alleles to build coexpression networks using the WGCNA package^91^. The soft power threshold was calculated as the first power to exceed a scale-free topology fit index of 0.9 for each network separately. Topographical overlap matrices (TOM) were calculated with the blockwiseModules function using TOMType = “unsigned”, and the minimum module size was set to 60. The parameter mergeCutHeight = 0.15 was used to merge similar modules. The threshold was calculated based on the pairwise distance between alleles. In brief, we calculated the Euclidean distance between the module eigengenes and used these values to calculate the distance of the alleles. When the distance between alleles was >50% of the median maximum distance between eigengenes, these alleles were classified as showing divergent expression patterns in different modules; otherwise, they were classified as having similar expression patterns.

### GO enrichment analysis

GO enrichment analysis was performed using the R package clusterProfiler with a *p* value of 0.05 and a *q* value of 0.05^92^.

### Identification of 6-gingerol biosynthesis pathway genes and phylogenetic analysis of shikimate O-hydroxycinnamoyltransferase enzyme (EC: 2.3.1.133) genes

The E2P2 package v3.1 (https://gitlab.com/rhee-lab/E2P2/tree/master) was used to obtain enzymatic annotations for coding genes. We mapped genes to 6-gingerol biosynthesis pathways by querying KEGG pathways. The identified shikimate O-hydroxycinnamoyltransferase enzyme (EC: 2.3.1.133) genes were subjected to phylogenetic analysis to determine their grouping pattern and genetic relationships. The phylogenetic relationships were constructed by maximum likelihood using IQ-TREE v1.6.7^80^ with the best-fit model (JTT + R5).

### Multilayered hierarchical gene regulatory network (ML-hGRN) construction

Pathways and biological processes are regulated in multilayered hierarchical gene regulatory networks (ML-hGRNs). The backward elimination random forest algorithm (BWERF) is an improved tool for constructing ML-hGRNs with gene expression data^93^. Expressed genes in the 6-gingerol biosynthetic pathway were selected to identify the TFs likely targeting these genes, and 1617 TFs were identified. Then, we constructed an ML-hGRN using a BWERF with 6-gingerol biosynthesis pathway genes as the bottom layer and the 1617 TFs in the regulatory layer^93^. A three-layer GRN was constructed based on 60 TFs potentially regulating 6-gingerol biosynthesis pathway genes either directly or indirectly. Finally, Cytoscape v3.7.1^92^ was used to visualize the network. Based on the gene regulatory network, the TFBSs in the promoter regions (2 kb upstream sequences) of the genes were used to identify the key regulatory TFs. If a TF could bind to the promoter region of any gene of the 6-gingerol biosynthesis pathway, it was considered a key TF.

## Supplementary information

Ginger_supplymentary_materials

## Data Availability

This Whole-Genome Shotgun Project data have been deposited in DDBJ/ENA/GenBank under the accession identifier JACMSC000000000. The version described in this paper is version JACMSC010000000. The raw sequence data have been deposited in the Short Read Archive under NCBI BioProject ID PRJNA647255.
