# Supplementary material for: Haplotype-resolved genome assembly and allele-specific gene expression in cultivated ginger
Source: Hortic Res. 2021 Aug 5;8:188. doi: 10.1038/s41438-021-00599-8 (PMC8342452; doi:10.1038/s41438-021-00599-8)
Supplement: Supplementary file 1 — Ginger_supplymentary_materials [file 41438_2021_599_MOESM1_ESM.docx]

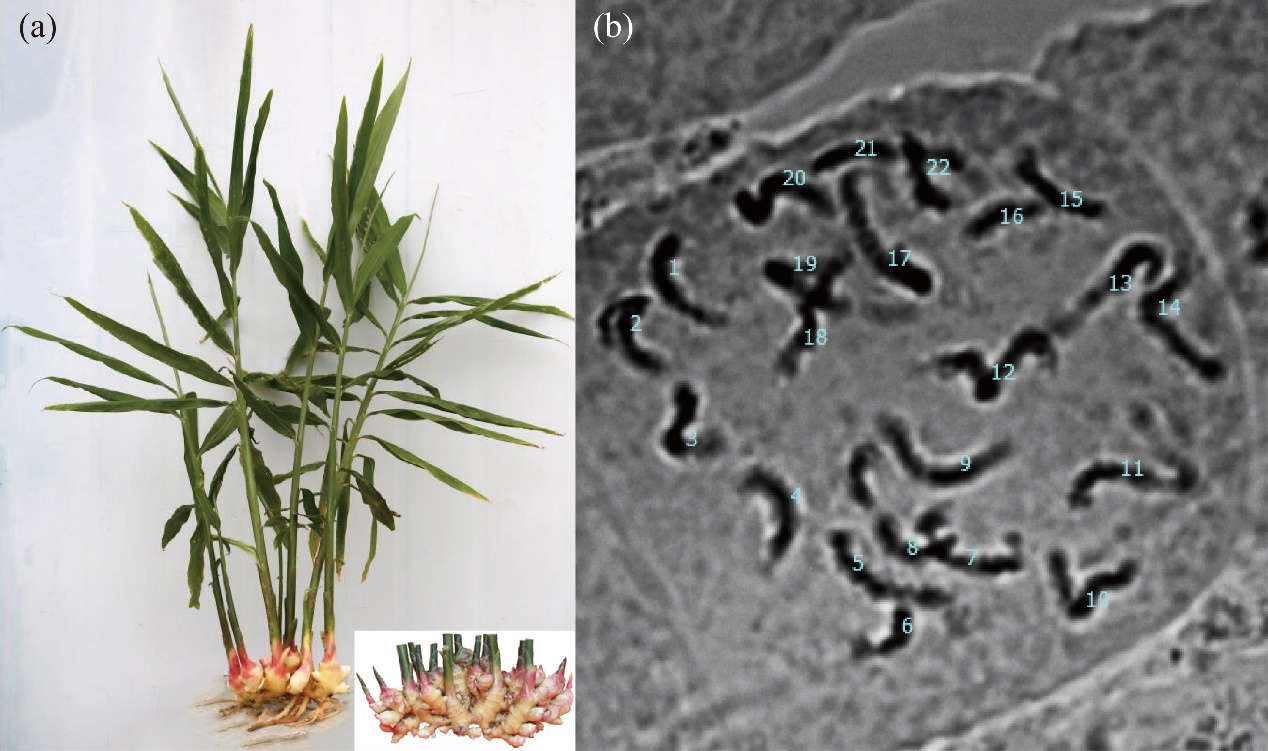


**Figure S1. Images of the seedling and mature rhizome of the sequenced individual and its mitotic metaphase chromosomes.** (a) Images of the whole seedling and its mature rhizome. (b) The chromosome number in mitotic metaphase was 22, indicating that the sequenced individual was diploid.


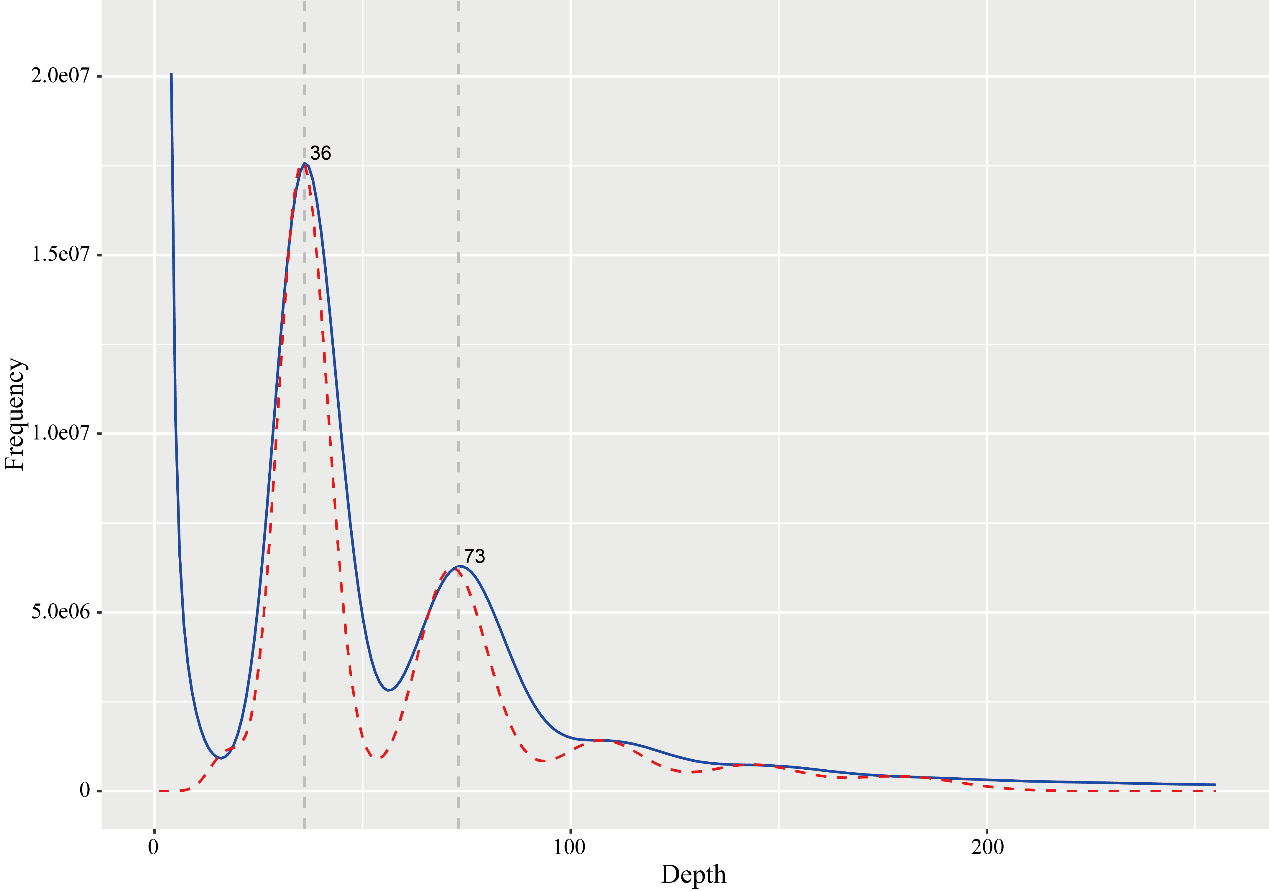


**Figure S2. The 17-mer distribution of Illumina short-read data.** A *K*-mer refers to an artificial sequence division of *K* nucleotides. Genomic characteristics (genome size and repeat structure) can be estimated based on *K*-mer frequencies.


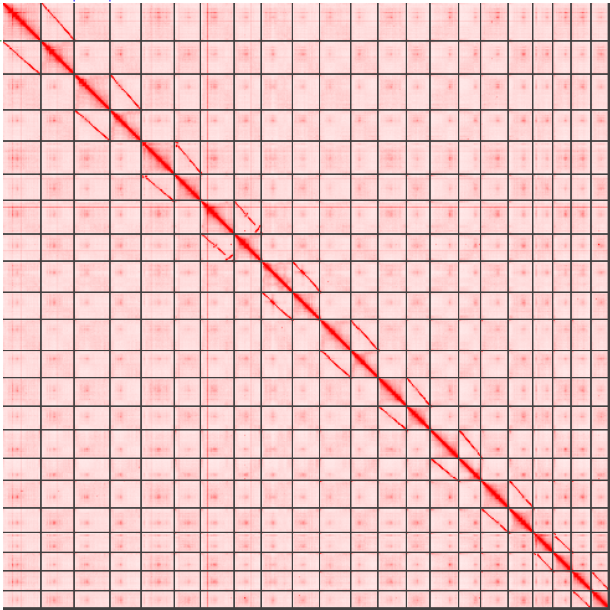


**Figure S3. Hi-C linkage density heat map of assemble contigs.**

**
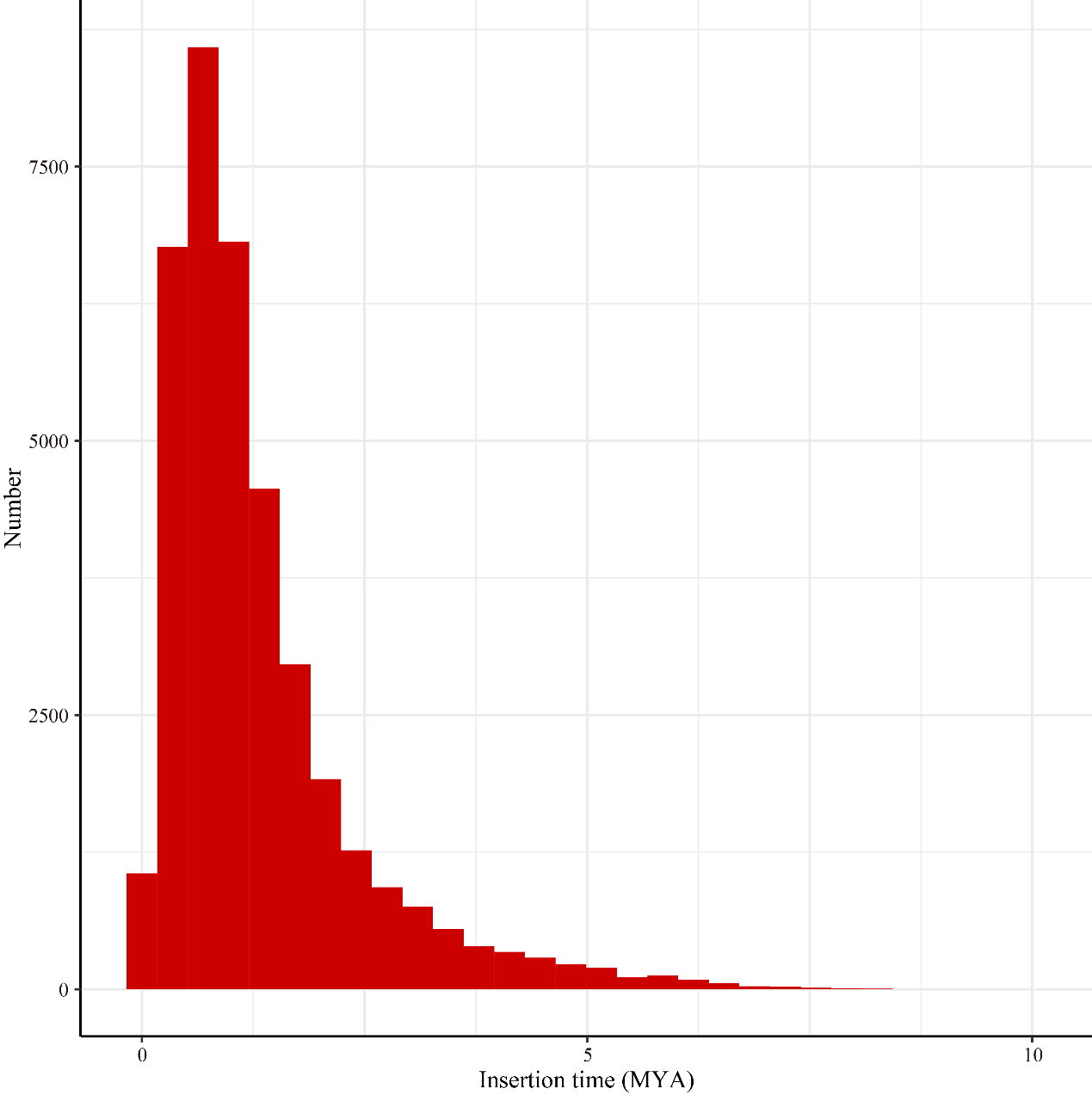
**

**Figure S4. Insertion time of long terminal repeat retrotransposons (LTR-RTs) in the ginger genome.**


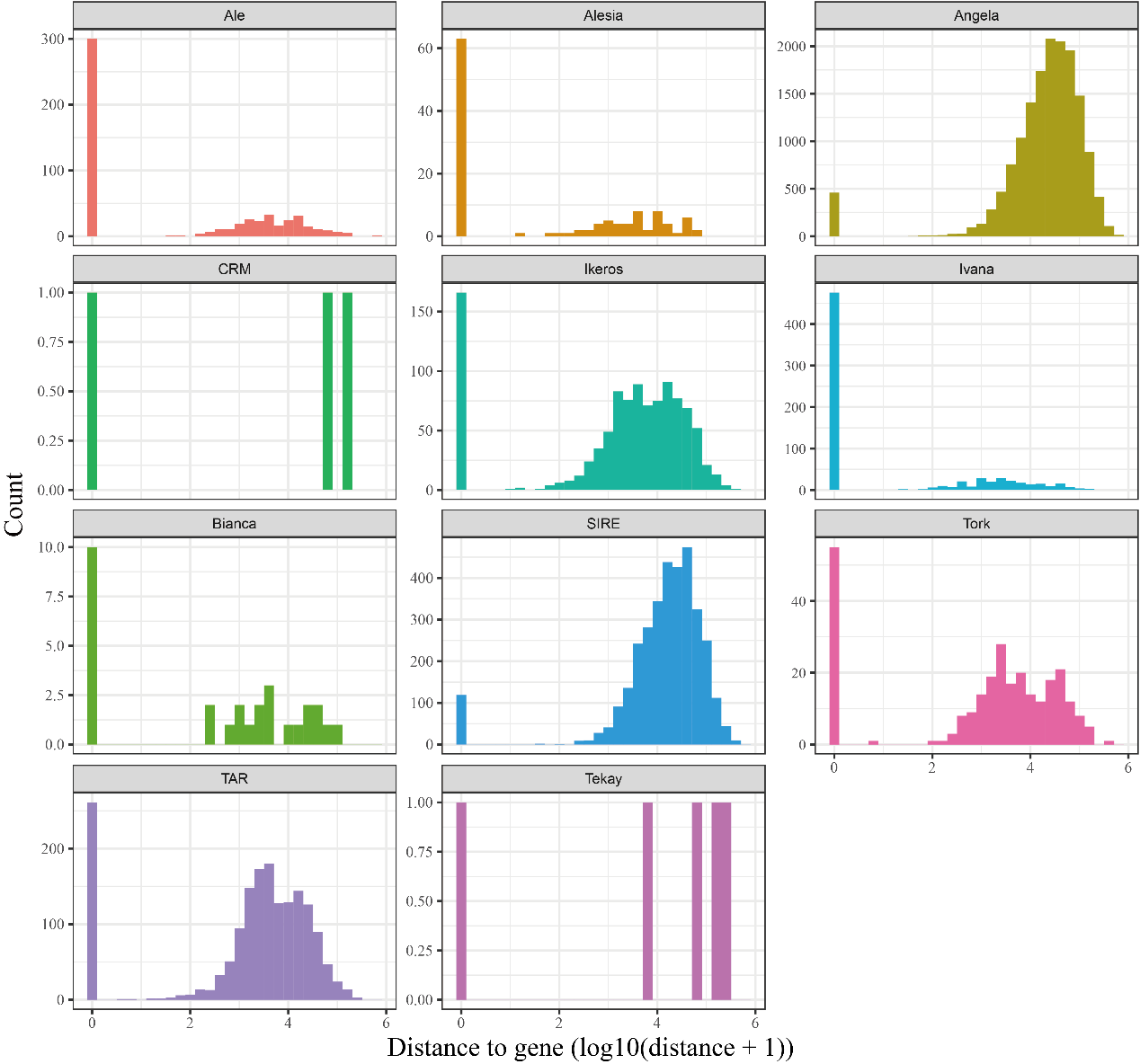


**Figure S5. Proliferation history of different subfamilies of the *Copia* class of long terminal repeat retrotransposons (LTR-RTs) in the ginger genome.**


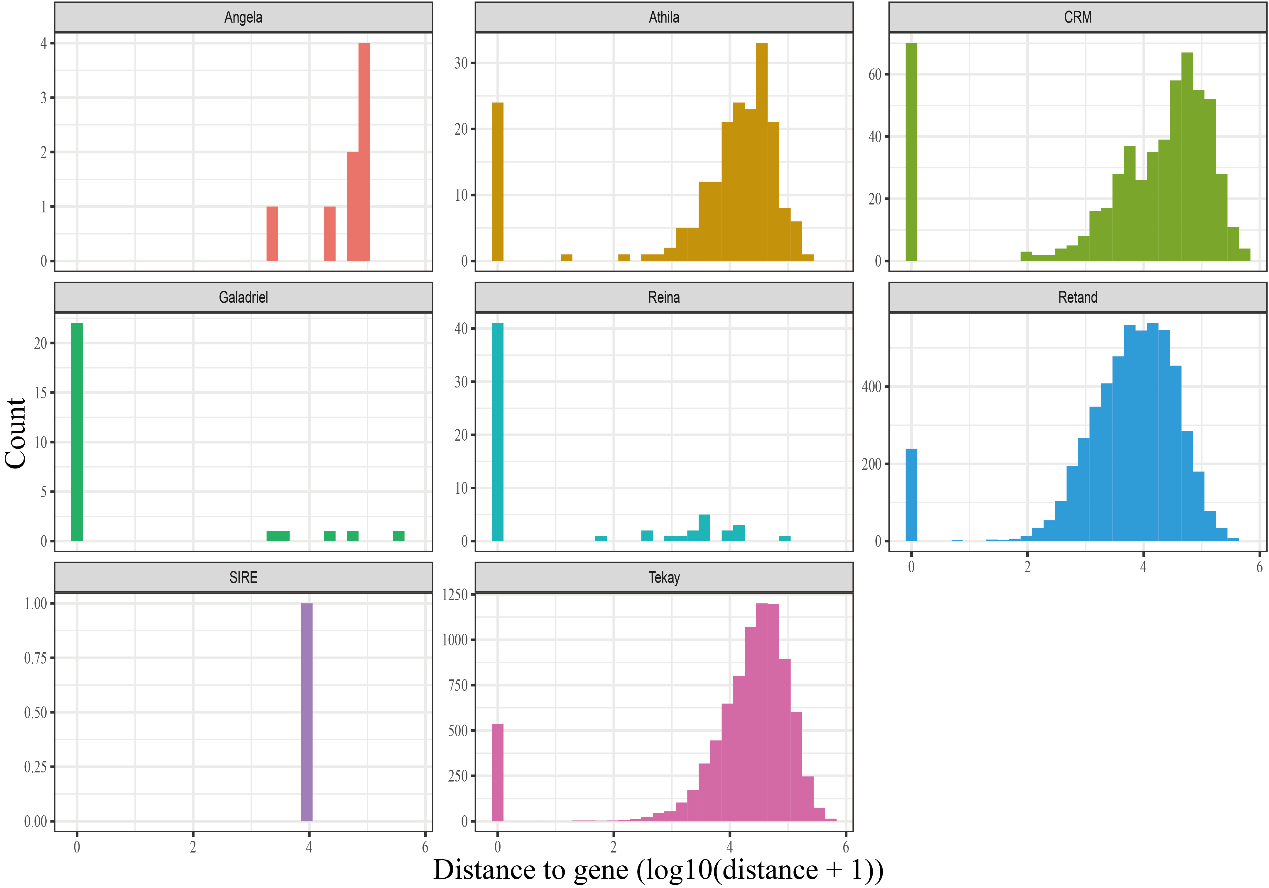


**Figure S6. Proliferation history of different subfamilies of the *Gypsy* class of long terminal repeat retrotransposons (LTR-RTs) in the ginger genome.**


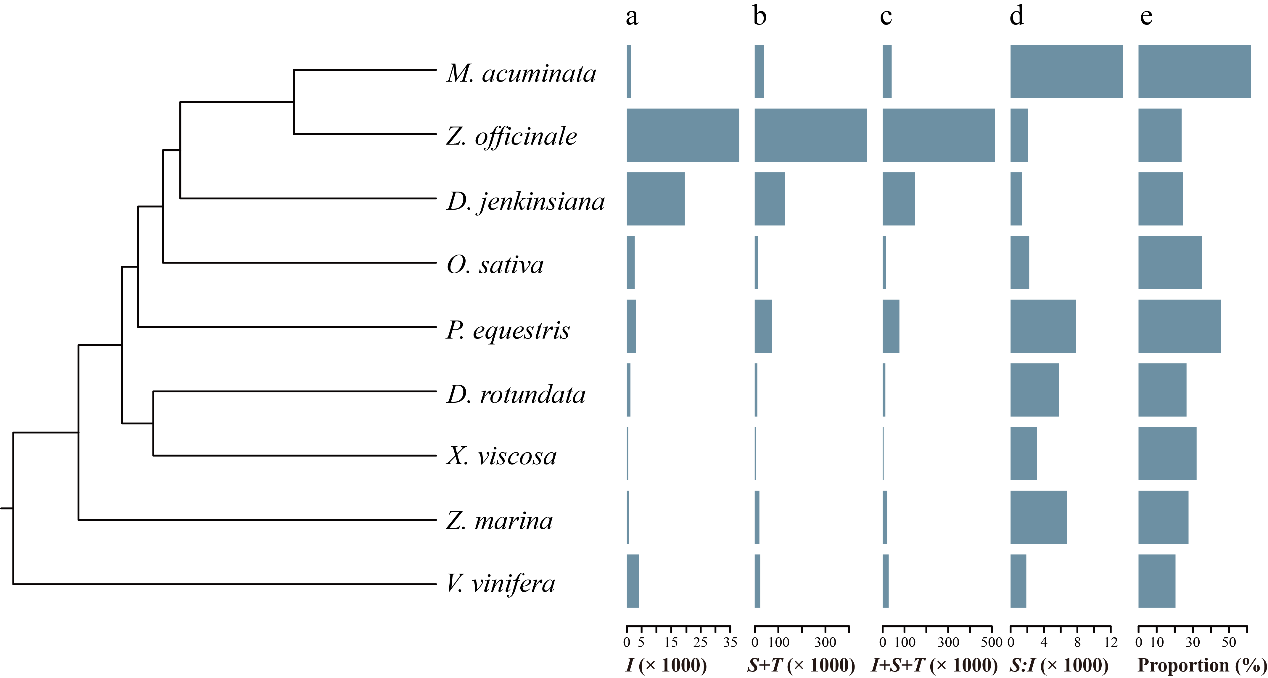


**Figure S7. Birth and death of long terminal repeat-retrotransposons (LTR-RTs) in the genome of ginger*.*** (a) Total numbers of LTR-RTs in the genome; (b) comparison of *S* + *T* values among plant species; *S*, number of solo-LTRs; *T*, number of truncated LTR-RTs. (c) Total numbers of intact LTR-RTs and traces of LTR-RT deaths; *I*, number of intact LTR-RTs. (d) ratios of solo-LTRs to intact LTR-RTs (*S*:*I*). (e) The proportions of LTR-RTs found in the clusters with high removal rates (filtered *S*:*I* ≥ 3).


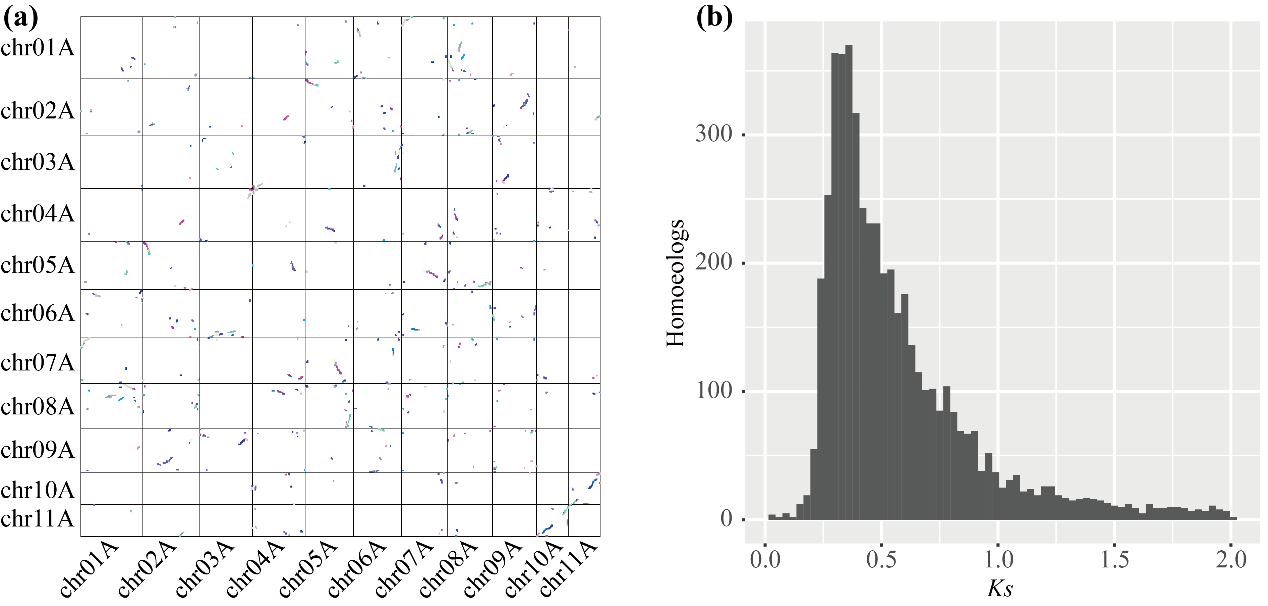


**Figure S8. Gene collinearity and *K_s_* distribution of ginger haplotype chromosome A.**


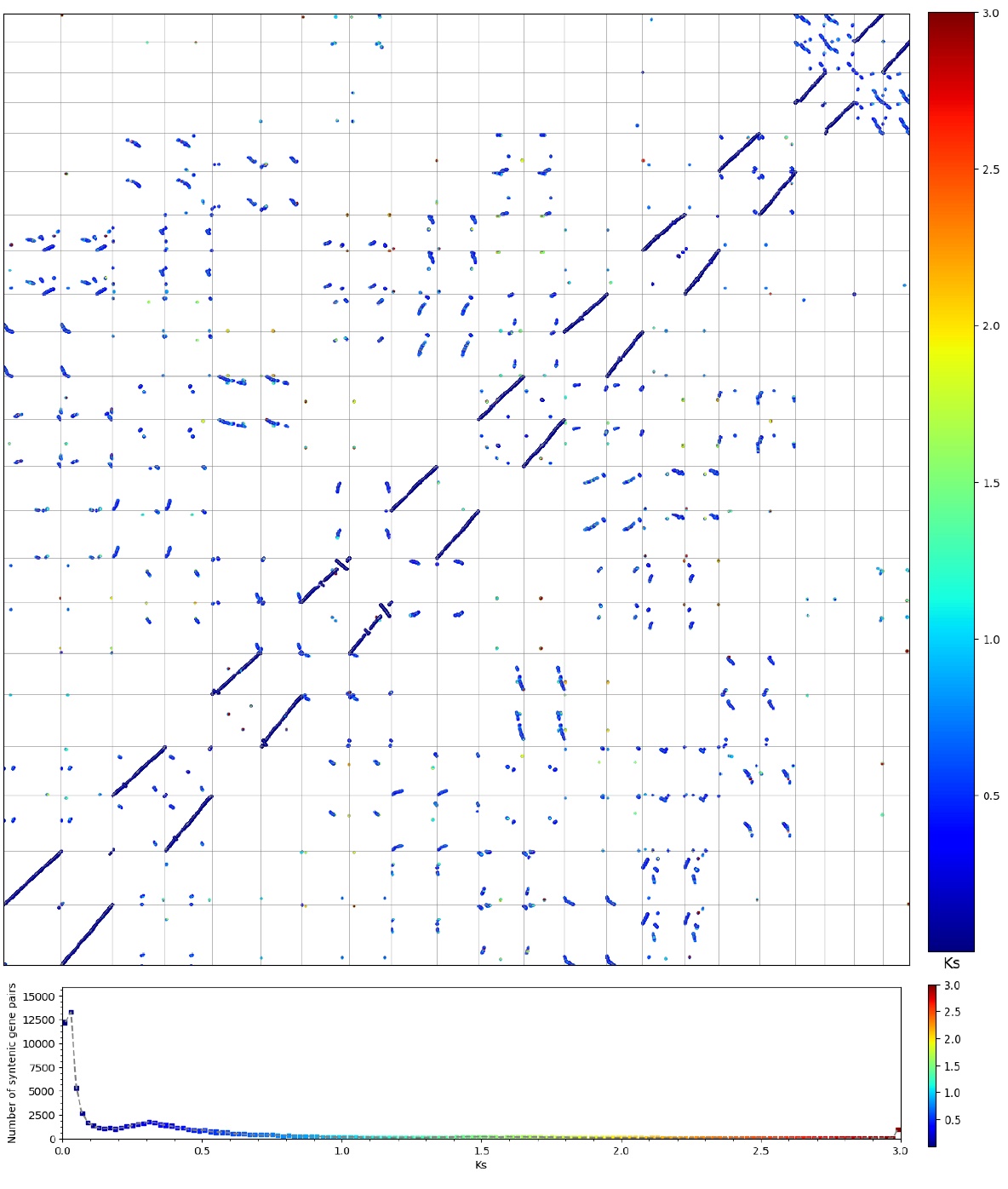


**Figure S9. Gene collinearity and *K_s_* distribution in ginger.**


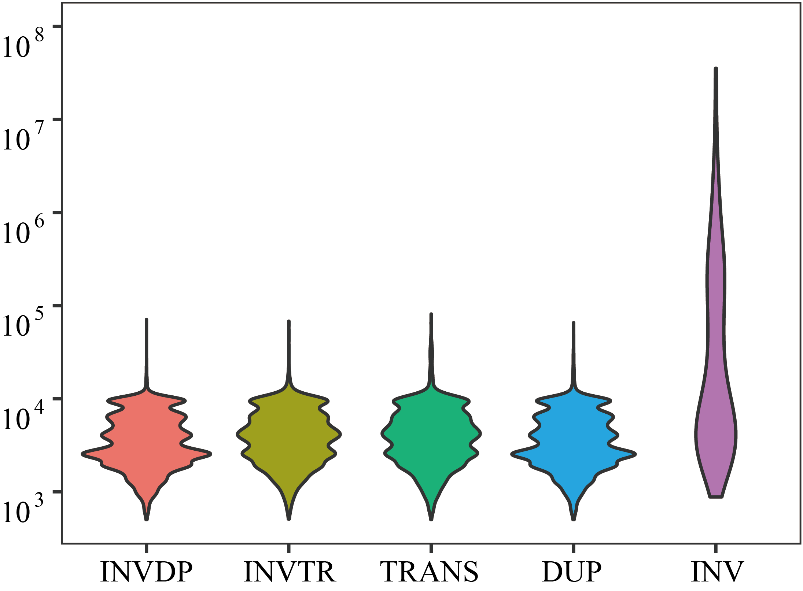


**Figure S10. Statistics of structural variation between allelic chromosome pairs.** Size distributions of different types of structural variation in the ginger genome. INVDP: inverted duplications, INVTR: inverted translocations, TRANS: translocations, DUP: duplications, INV, inversions.


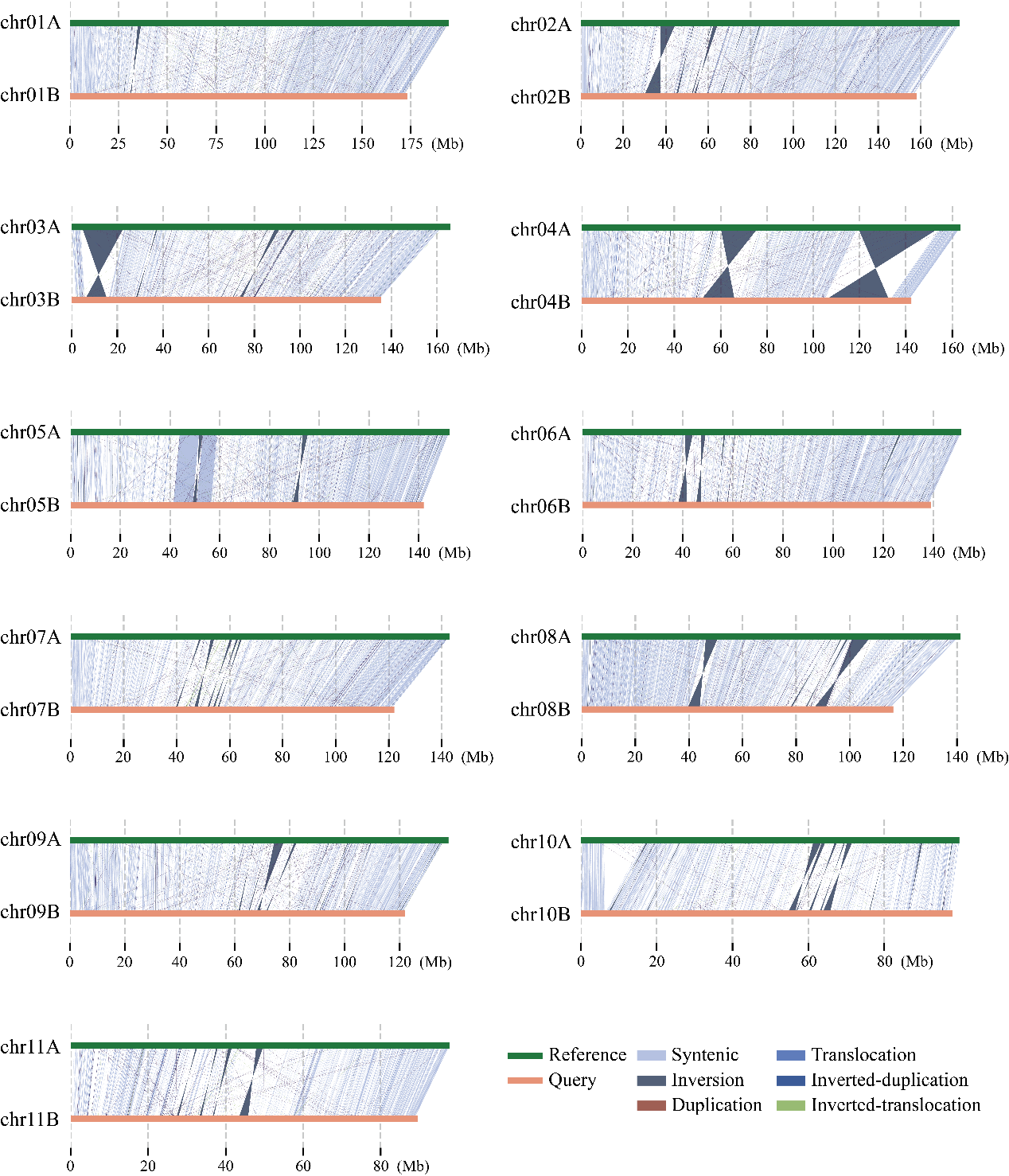


**Figure S11. Visualization of structural variation between allelic chromosome pairs.**


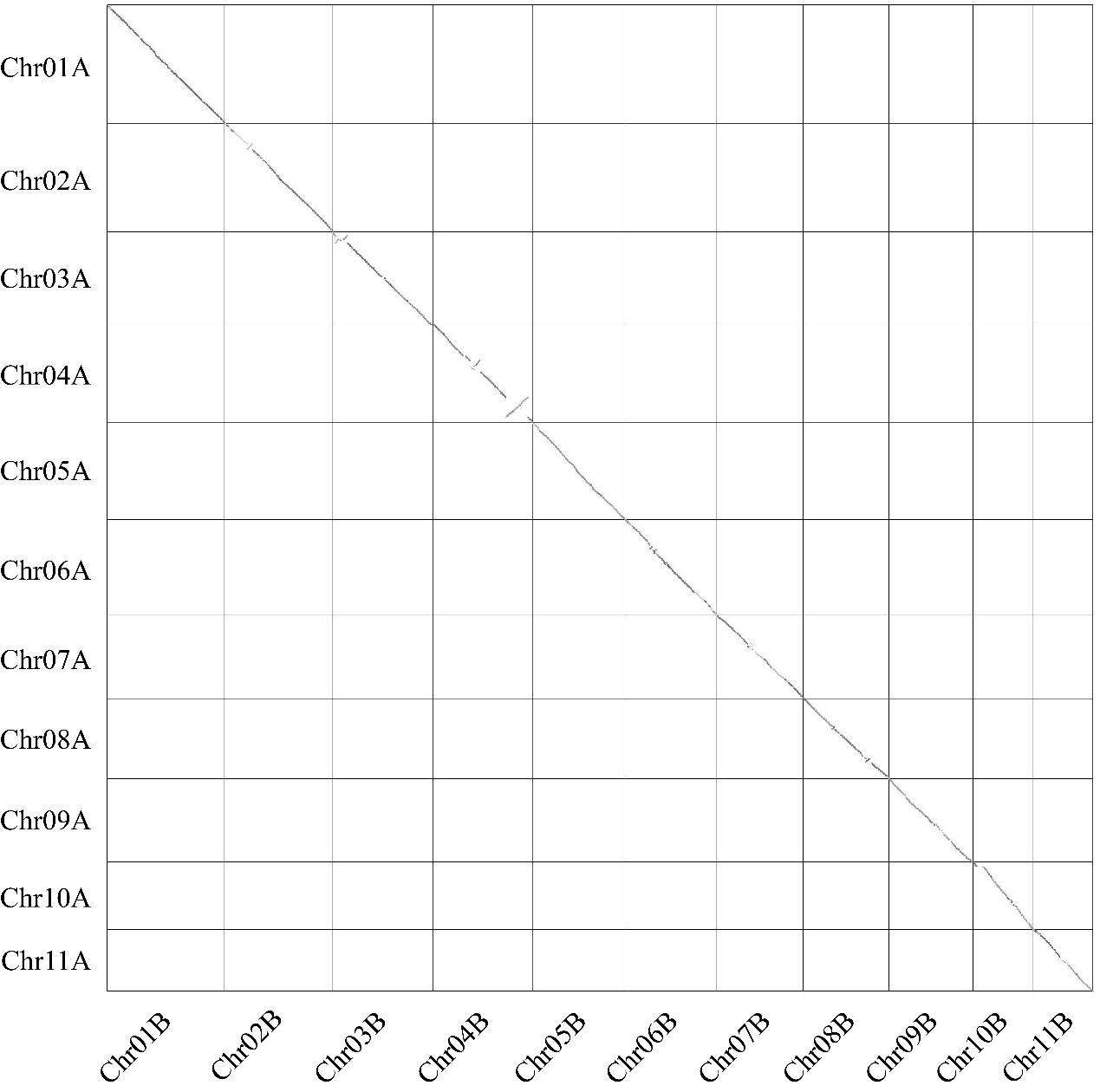


**Figure S12. Dot plots of identified alleles along allelic chromosome pairs in the ginger genome.**

**
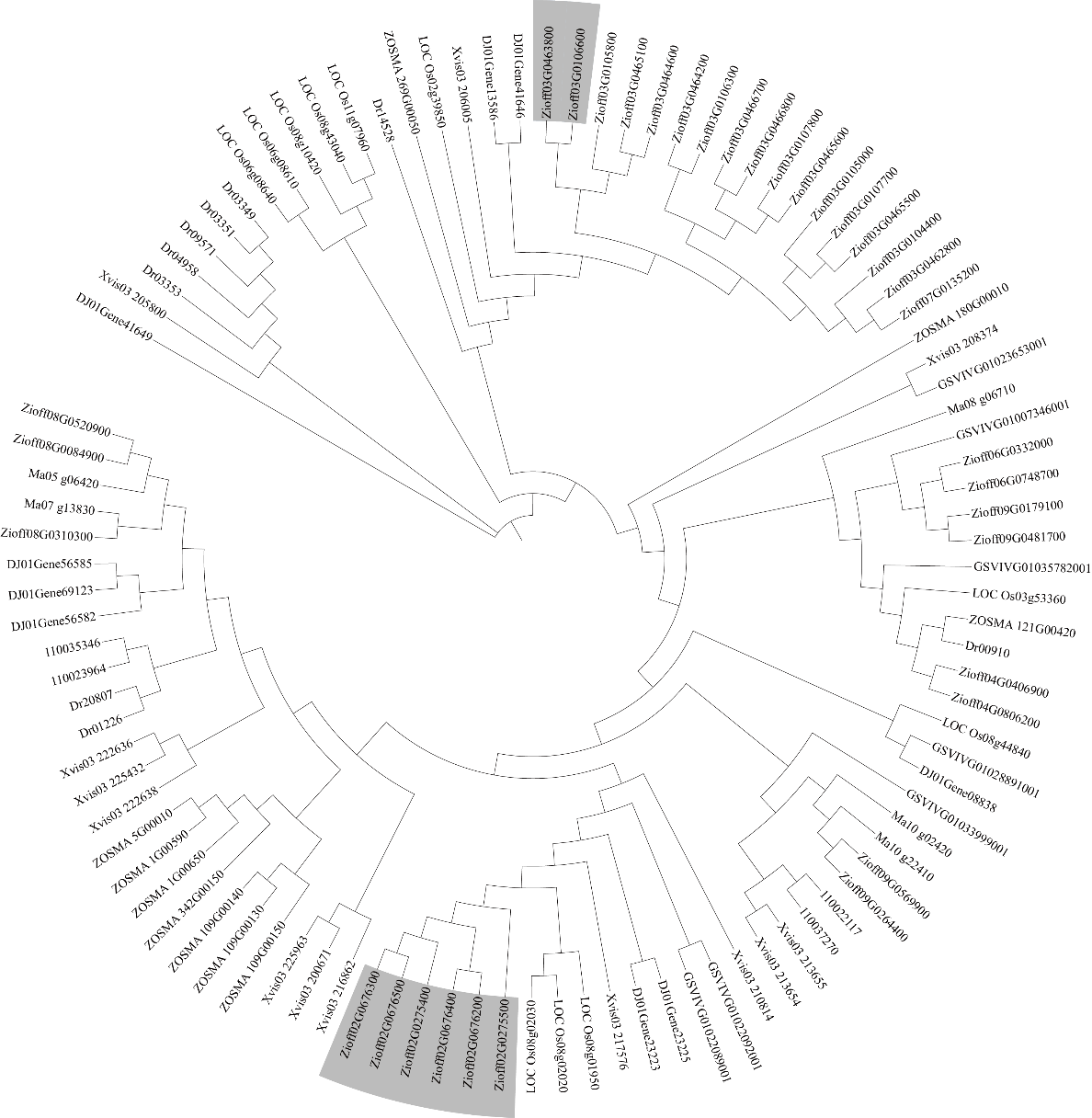
**

**Figure S13. Phylogenetic analysis of shikimate O-hydroxycinnamoyltransferase enzyme (EC: 2.3.1.133) genes using the full sequences of 9 species (*M. acuminata*, *D. jenkinsiana*, *O. sativa*, *X. viscosa*, *P. equestris*, *D. rotundata*, *Z. marina*, *V. vinifera* and ginger). The genes with a gray background are responsible for key steps in ginger.**

**Table S1. DNA-seq and RNA-seq data.**

**Table S2. Statistics of the ONT datasets.**

**Table S3.** **Statistics of assemblies with different methods.**

**Table S4. Statistics of preliminary assemblies.**

**Table S5. Chromosome length in the final ginger genome assembly.**

**Table S6. Summary of BUSCO evaluation results for the final ginger genome assembly.**

**Table S7. Summary of RNAs in the ginger genome annotation.**

**Table S8. Functional annotation of the predicted ginger genes.**

**Table S9. Repetitive DNA sequences in the ginger genome.**

**Table S10. Summary of structural variations between allelic chromosome pairs.**

**Table S11. Summary of structural variations in the ginger genome.**

**Table S12. Summary of differences in allelic gene expression in the ginger genome.**

**Table S13. GO enrichment of stably expressed alleles in the ginger genome.**

**Table S14. GO enrichment of dynamically expressed alleles in the ginger genome.**

**Table S15. Allelic gene coexpression in the ginger genome.**

**Table S16. GO enrichment of divergent expression alleles in the ginger genome.**

**Table S17. Important transcription factor genes involved in 6-gingerol biosynthesis in ginger.**

| **Genes** | **Gene families** | **Homoeologous gene in *Arabidopsis thaliana*** | **E-value** | **Description** |
| --- | --- | --- | --- | --- |
| Zioff02G0663800 | ERF | AT1G53910.1 | 4.00E-42 | related to AP2 12 |
| Zioff03G0112100 | ERF | AT1G53910.1 | 4.00E-44 | related to AP2 12 |
| Zioff01G0655000 | bHLH | AT1G59640.1 | 4.00E-62 | BIG PETAL P |
| Zioff02G0231600 | HD-ZIP | AT3G01470.1 | 5.00E-50 | homeobox 1 |
| Zioff02G0634100 | HD-ZIP | AT3G01470.1 | 1.00E-52 | homeobox 1 |
| Zioff11G0166700 | HD-ZIP | AT3G01470.1 | 2.00E-56 | homeobox 1 |
| Zioff05G0128800 | GATA | AT3G60530.1 | 1.00E-56 | GATA transcription factor 4 |
| Zioff05G0501300 | GATA | AT3G60530.1 | 9.00E-56 | GATA transcription factor 4 |
| Zioff01G0512700 | WRKY | AT4G18170.1 | 4.00E-43 | WRKY DNA-binding protein 28 |
| Zioff02G0636100 | bZIP | AT5G10030.1 | 1.00E-158 | TGACG motif-binding factor 4 |
| Zioff01G0795400 | ERF | AT5G18450.1 | 2.00E-39 | ERF family protein |
| Zioff05G0491000 | BBR-BPC | AT5G42520.1 | 1.00E-113 | basic pentacysteine 6 |
| Zioff09G0391700 | Trihelix | AT1G76890.2 | 3.00E-61 | Trihelix family protein |
